# Altering cold-regulated gene expression decouples the salicylic acid–growth trade-off in Arabidopsis

**DOI:** 10.1093/plcell/koae210

**Published:** 2024-07-26

**Authors:** María A Ortega, Rhodesia M Celoy, Francisco Chacon, Yinan Yuan, Liang-Jiao Xue, Saurabh P Pandey, MaKenzie R Drowns, Brian H Kvitko, Chung-Jui Tsai

**Affiliations:** Warnell School of Forestry and Natural Resources, University of Georgia, Athens, GA 30602, USA; Department of Genetics, University of Georgia, Athens, GA 30602, USA; Department of Plant Biology, University of Georgia, Athens, GA 30602, USA; Warnell School of Forestry and Natural Resources, University of Georgia, Athens, GA 30602, USA; Department of Genetics, University of Georgia, Athens, GA 30602, USA; Warnell School of Forestry and Natural Resources, University of Georgia, Athens, GA 30602, USA; Department of Genetics, University of Georgia, Athens, GA 30602, USA; College of Forest Resources and Environmental Science, Michigan Technological University, Houghton, MI 49931, USA; Warnell School of Forestry and Natural Resources, University of Georgia, Athens, GA 30602, USA; Department of Genetics, University of Georgia, Athens, GA 30602, USA; State Key Laboratory of Tree Genetics and Breeding, College of Forestry, Nanjing Forestry University, Nanjing, Jiangsu 210037, China; Warnell School of Forestry and Natural Resources, University of Georgia, Athens, GA 30602, USA; Department of Genetics, University of Georgia, Athens, GA 30602, USA; Department of Plant Biology, University of Georgia, Athens, GA 30602, USA; Warnell School of Forestry and Natural Resources, University of Georgia, Athens, GA 30602, USA; Department of Genetics, University of Georgia, Athens, GA 30602, USA; Department of Plant Biology, University of Georgia, Athens, GA 30602, USA; Department of Plant Pathology, University of Georgia, Athens, GA 30603, USA; Warnell School of Forestry and Natural Resources, University of Georgia, Athens, GA 30602, USA; Department of Genetics, University of Georgia, Athens, GA 30602, USA; Department of Plant Biology, University of Georgia, Athens, GA 30602, USA

## Abstract

In Arabidopsis (*Arabidopsis thaliana*), overproduction of salicylic acid (SA) increases disease resistance and abiotic stress tolerance but penalizes growth. This growth–defense trade-off has hindered the adoption of SA-based disease management strategies in agriculture. However, investigation of how SA inhibits plant growth has been challenging because many SA-hyperaccumulating Arabidopsis mutants have developmental defects due to the pleiotropic effects of the underlying genes. Here, we heterologously expressed a bacterial SA synthase gene in Arabidopsis and observed that elevated SA levels decreased plant growth and reduced the expression of cold-regulated (*COR*) genes in a dose-dependent manner. Growth suppression was exacerbated at below-ambient temperatures. Severing the SA-responsiveness of individual *COR* genes was sufficient to overcome the growth inhibition caused by elevated SA at ambient and below-ambient temperatures while preserving disease- and abiotic-stress-related benefits. Our results show the potential of decoupling SA-mediated growth and defense trade-offs for improving crop productivity.

## Teaser

Constitutive expression of individual cold-regulated genes can override salicylic acid-induced growth inhibition while maintaining enhanced disease resistance.

## Introduction

The phytohormone salicylic acid (SA) has well-established roles in immune signaling in plants (Vlot et al. 2009; Peng et al. 2021; Ullah et al. 2023). Considerable advances in understanding SA-mediated defense mechanisms have been made possible through forward genetic screens in the model plant Arabidopsis (*Arabidopsis thaliana*). These screens have identified mutants with enhanced disease resistance resulting from increased SA accumulation (Heil and Baldwin 2002; Rivas-San Vicente and Plasencia 2011). Examples include *constitutive expresser of pathogenesis-related genes5* (*cpr5*) (Bowling et al. 1997), *defense no death1* (*dnd1*) (Yu et al. 1998), *sap and miz1 domain-containing ligase1* (*siz1*) (Lee et al. 2007), and *suppressor of rps4-RLD1* (*srfr1*) (Kwon et al. 2004). However, SA-elevated mutants exhibit reduced growth, sometimes in a temperature-dependent manner (Heidel et al. 2004; Wang et al. 2009; Alcázar and Parker 2011; Huot et al. 2014; Albrecht and Argueso 2017).

Mechanistic research into the growth–defense trade-off has been challenging, in part because SA regulates various physiological and developmental processes in its own right (Rivas-San Vicente and Plasencia 2011; Peng et al. 2021). Such efforts are further complicated by the reliance on the so-called autoimmune or lesion mimic mutants which display constitutive activation of defense responses (including elevated SA) at the expense of plant growth and development (Bruggeman et al. 2015; van Wersch et al. 2016). These autoimmune mutants have diverse origins, as their mutated genes are involved in not only immune signaling but also programmed or induced cell death, second messengers, hormonal pathways, or other cellular and subcellular processes (Bruggeman et al. 2015; van Wersch et al. 2016; Freh et al. 2022). This makes it difficult to isolate the specific effects of SA on growth. For instance, *cpr5* mutants exhibit juvenile leaf senescence in additional to dwarfism (Bowling et al. 1997) and CPR5 was later shown to have dual function as a nucleoporin associated with the nuclear pore complex (Gu et al. 2016) and an RNA-binding protein associated with RNA processing complexes (Peng et al. 2022). The *dnd1* mutant shows severe dwarfism and harbors a loss-of-function allele encoding CYCLIC NUCLEOTIDE-GATED CATION CHANNEL2 (CNGC2) with roles in Ca^2+^ signaling associated with not only defense but also plant development (Köhler et al. 1999; Clough et al. 2000; Chan et al. 2003; Ma et al. 2010). *SIZ1* encodes a small ubiquitin-like modifier E3 ligase and its mutant phenotypes include altered chloroplast functions and prolific bolting (Miura et al. 2013; Kong et al. 2017). *SRFR1* encodes a tetratricopeptide repeat domain-containing protein that functions as a negative immune regulator via its interactions with disease resistance proteins as well as transcription factors involved in developmental processes (Kim et al. 2010; Li et al. 2010; Bhattacharjee et al. 2011; Kim et al. 2014). Disentangling the complex interplay between growth and SA-mediated defense calls for development of an experimental system to directly manipulate SA biosynthesis in plants without the confounding influence of pleiotropic gene mutations.

Several approaches have been used to augment SA biosynthesis in plants with varying degrees of success. In tobacco (*Nicotiana tabacum*), constitutive co-expression of *Escherichia coli entC* and *Pseudomonas fluorescens pmsB* genes encoding isochorismate synthase (ICS) and isochorismate pyruvate lyase (IPL), respectively, resulted in elevated SA accumulation and enhanced disease resistance when the enzymes were targeted to the chloroplasts (Verberne et al. 2000). However, a similar approach in Arabidopsis expressing a chloroplast-targeted fusion gene of *pchA* (*ICS*) and *pchB* (*IPL*) from *Pseudomonas aeruginosa* led to severe dwarfism and sterility (Mauch et al. 2001). This has discouraged further research with SA bioengineering in Arabidopsis. In poplars (*Populus tremula* × *Populus alba* and *Populus nigra*), SA hyperaccumulation resulting from overexpression of *Yersinia enterocolitica Irp9* encoding a bifunctional SA synthase targeted to the chloroplasts enhanced abiotic stress tolerance and rust resistance without affecting plant growth as in tobacco (Xue et al. 2013; Ullah et al. 2022). The inconsistent results in Arabidopsis may reflect taxon-specific sensitivity to SA, although many autoimmune mutants with similar SA increases did not exhibit the same phenotypic severity or sterility (Bowling et al. 1997; Clough et al. 2000; Mauch et al. 2001; Lee et al. 2007; Kim et al. 2010). Alternatively, other experimental variations might have contributed, which justifies an independent reexamination.

In this study, we sought to augment plastidial SA biosynthesis in Arabidopsis by adopting the poplar strategy expressing a bacterial bifunctional SA synthase *Irp9* (Xue et al. 2013). We successfully obtained viable transgenic lines with a broad range of SA levels to directly assess the effects of SA on growth. We then used these lines to identify an inhibitory role of SA on *COLD-REGULATED* (*COR*) gene expression. This SA inhibition interfered with multiple COR-associated functions, including leaf longevity and low temperature responses, which culminated in reduced growth especially at below-ambient temperatures. We provide evidence to suggest that the SA-mediated trade-off can be circumvented by transcriptional rewiring of *CORs* to balance growth and defense.

## Results

### Elevated SA in transgenic Arabidopsis reduces growth in a dose-dependent manner

**Figure 1.**
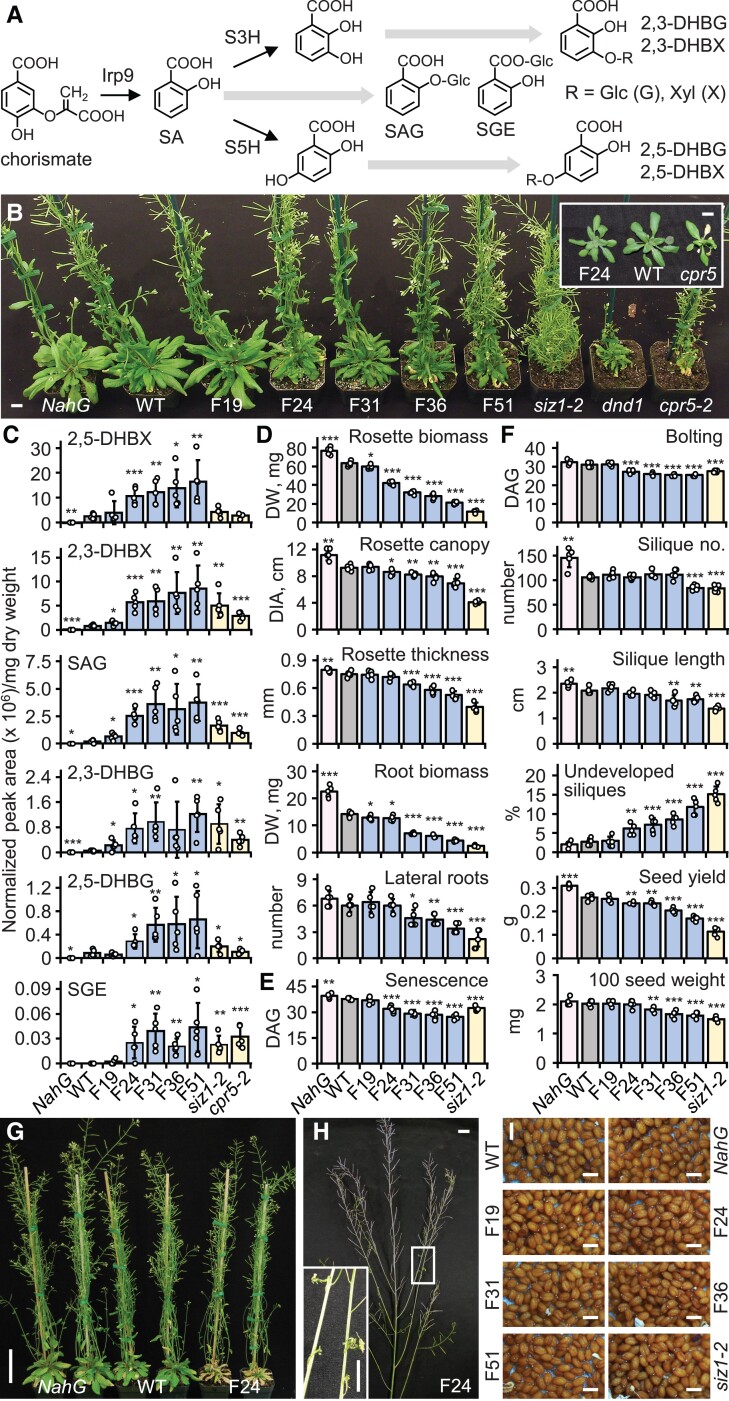
Characterization of the Arabidopsis *Fd-Irp9-OE* lines. **A)** Simplified diagram of Irp9-mediated SA biosynthesis and downstream catabolism and conjugation. **B)** Representative plant morphology 45 DAG at 22 °C. Inset shows juvenile senescence of *cpr5-2* not seen in F24. Scale bars, 1 cm. **C)** Levels of SA-derived conjugates in rosette leaves of WT, homozygous *Fd-Irp9*-OE lines, and autoimmune mutants at bolting. Data are means ± SD of *n* = 5 independent pools, each sampled from 3 rosette leaves per plant. **D–F)** Vegetative growth traits at 28 DAG (D), onset of senescence (E) and bolting (F), and seed traits at 50 DAG (F). Data are means ± SD of *n* = 5 plants. **G)** Premature senescence in F24 (right) compared with WT (middle) and *NahG* (left). Scale bar, 5 cm. **H)** Representative images of F24 at seed set (scale bar, 1 cm). Inset shows incompletely developed siliques (scale bar, 0.5 cm). **I)** Representative images of seeds. Scale bars, 0.5 mm. Statistical significance was determined by 2-sided Student's *t*-test against WT (***P < 0.001; **P < 0.01; *P < 0.05). The growth phenotypes were reproducible in more than 10 experiments. The experiment for SA analysis was conducted 4 times with similar trends. DHBG, dihydroxybenzoate glucoside; DHBX, dihydroxybenzoate xyloside; SA, salicylic acid; S3H, SA 3-hydroxylase; S5H, SA 5-hydroxylase; SAG, SA-glucoside; SGE, SA-glucose ester.

We generated transgenic Arabidopsis plants overexpressing *Irp9*, a bacterial bifunctional SA synthase gene, with a ferredoxin chloroplast-targeting signal (Xue et al. 2013), named here *Fd-Irp9-*OE lines. We selected five independent lines with different levels of SA accumulation and growth phenotypes for characterization (Fig. 1). The SA-deficient transgenic *NahG* plants expressing a bacterial SA hydroxylase (Reuber et al. 1998) were included as reference. Four *Fd-Irp9-*OE lines (F24, F31, F36, and F51) had SA-derivative levels that approached or surpassed those detected in the autoimmune mutants, whereas line F19 was similar to the Col-0 wild type (WT) (Fig. 1, A–C, and Supplementary Fig. S1). Hereafter, we refer to F24, F31, F36, and F51 as high-SA (hiSA) lines. The SA-related metabolites we detected are similar to those reported to increase in senescing or pathogen-challenged Arabidopsis leaves, or in mutants or natural accessions with elevated SA (Bartsch et al. 2010; Li et al. 2014) (Supplementary Fig. S2). SA-glucoside (SAG) and dihydroxybenzoate xylosides (2,3-DHBX and 2,5-DHBX) were by far the most abundant SA-derivatives in the hiSA lines, followed by dihydroxybenzoate glucosides (2,3-DHBG and 2,5-DHBG), with SA-glucose ester (SGE) detected at low levels (Fig. 1C and Supplementary Fig. S1). For simplicity, we refer to these SA-derived compounds as SA metabolites, and their summed abundance as total SA levels.

The increased accumulation of SA metabolites negatively affected hiSA plant growth in a dose-dependent manner under long-day (16 h light) conditions at 22 °C (Fig. 1, B–D). In the hiSA lines, we did not observe the developmental abnormalities associated with autoimmune mutants, such as prolific bolting (*siz1-2*), severe dwarfism (*dnd1*), or juvenile senescence (*cpr5-2*) (Fig. 1B and inset). At 28 days after germination (DAG) of homozygous *Fd-Irp9*-OE lines, multiple growth parameters, including rosette size (length and thickness), rosette biomass, lateral root number, and root biomass, were all inversely associated with total SA levels (Fig. 1D). Line F51 was the most severely affected, followed in order by F36, F31, and F24; F19 was phenotypically indistinguishable from the WT. The hiSA lines reached the reproductive phase (bolting) 4–6 days earlier than the WT and displayed accelerated senescence, with apparent leaf yellowing occurring 5–10 days before that in the WT (Fig. 1, E–G). Accordingly, hiSA plants had shorter life spans and lower seed yields due to many undeveloped siliques as well as slightly reduced seed weight (Fig. 1, F–H), although their fully developed seeds appeared normal (Fig. 1I). These results demonstrate an inhibitory effect of SA on growth and support previous findings on the role of SA in leaf senescence and seed yield (Morris et al. 2000; Abreu and Munné-Bosch 2009; Zhang et al. 2013).

### Transgenic hiSA plants exhibit enhanced disease resistance and abiotic stress tolerance

**Figure 2.**
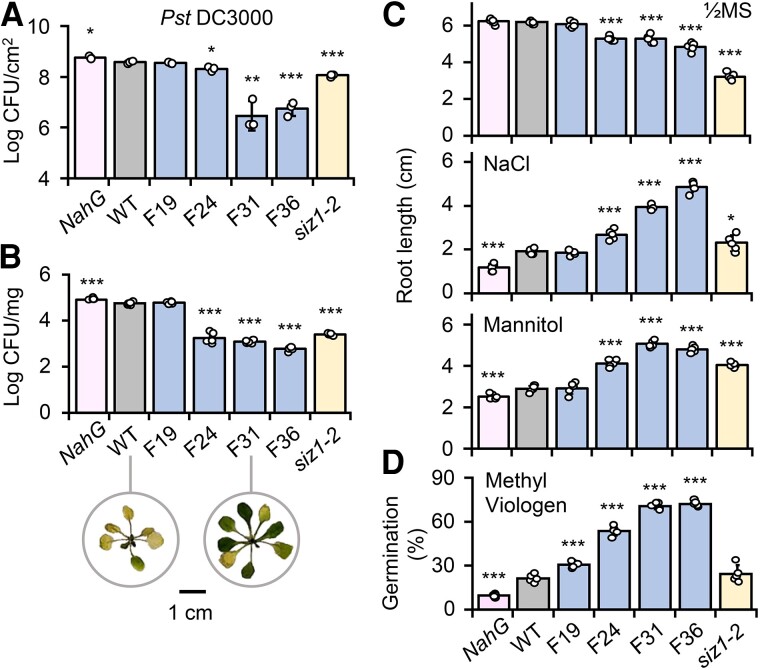
Responses of *Fd-Irp9-OE* lines to pathogen or abiotic stresses. **A)***Pst* DC3000 bacterial growth based on leaf infiltration of soil-grown plants at bolting. Data are means ± SD of *n* = 3 independent pools of 4 leaf discs sampled from 2 rosette leaves per plant. The experiment was performed 6 times with similar trends. **B)***Pst* DC3000 bacterial growth based on flood inoculation of 2-week-old seedlings in tissue culture. Representative plant images from WT and F31 three days after inoculation are shown. Scale bar, 1 cm. Data are means ± SD of *n* = 5 independent pools of 3 rosette leaves per plant. The experiment was performed twice with similar patterns. **C)** Primary root growth of 11-day-old seedlings on half-strength MS medium (1/2 MS) alone or with NaCl or mannitol. Data are means ± SD of *n* = 5 independent pools of 2 seedlings per pool (each data point was averaged from 2 seedlings). **D)** Seed germination in the presence of methyl viologen. Data are means ± SD of *n* = 5 independent pools of 10 seeds per pool. The abiotic stress experiments were performed twice with similar results. Statistical significance was determined by 2-sided Student's *t*-test against WT (***P < 0.001; **P < 0.01; *P < 0.05).

We assessed resistance to *Pseudomonas syringae* pv. tomato strain DC3000 (*Pst* DC3000) by both syringe infiltration of soil-grown plants and flood inoculation of in vitro-cultured seedlings (F51 was excluded because of low seed yield) (Fig. 2). We observed a negative association between leaf SA levels and bacterial growth 3 days post-inoculation (DPI), with significantly lower bacterial growth in hiSA lines (Fig. 2, A and B). These resistant lines showed mild disease symptoms and continued to produce rosette leaves under the in vitro assay conditions, whereas WT, F19, and *NahG* lines succumbed to *Pst* DC3000 during the monitoring period (Fig. 2B).

We also examined seedling sensitivity to abiotic stress treatments in vitro. Untreated F24, F31, and F36 seedlings had significantly shorter primary roots than the WT (Fig. 2C), consistent with the reduced root biomass of soil-grown plants (Fig. 1D). However, the reverse was true for seedlings grown on salt- or mannitol-containing medium (Fig. 2C). When seeds were sown on medium containing herbicidal methyl viologen, all four *Fd-Irp9*-OE lines, including F19, showed significantly higher germination rates than the WT (Fig. 2D). We noted that this SA-mediated protection against methyl viologen-induced oxidative stress was partially lost in *siz1-2* (Fig. 2D), highlighting the pleiotropic effects of the *siz1* mutation (Kim et al. 2021). Taken together, our results demonstrate a functional dichotomy for elevated SA biosynthesis in Arabidopsis; SA imposed a growth penalty under normal conditions but enhanced disease resistance and abiotic stress tolerance under adverse conditions.

### Transgenic hiSA plants show constitutive expression of systemic acquired resistance marker genes but repression of COR genes under nonstress conditions

**Figure 3.**
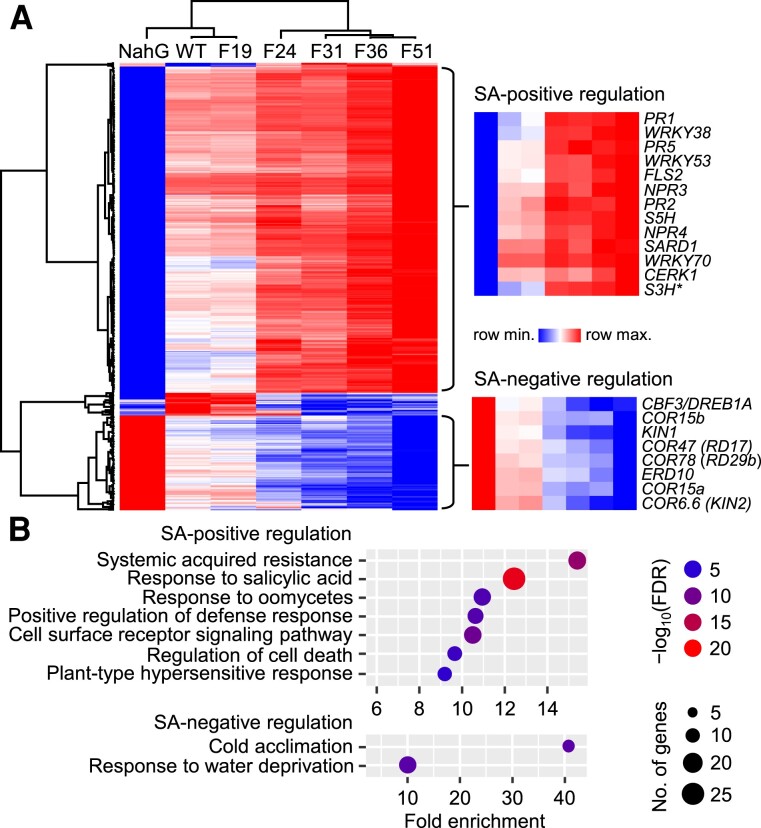
Leaf transcriptomic responses of the *Fd-Irp9*-OE lines. **A)** Hierarchical clustering of 471 SA-responsive genes selected according to differential expression in both F51 and *NahG* lines relative to the WT. Representative genes that were positively or negatively regulated by SA are shown on the right (*S3H not captured in the list was included for reference). Data were obtained from *n* = 4 independent pools, each sampled from 3 rosette leaves per plant at 30 DAG, except for F51 where *n* = 3 independent pools. Differential expression relative to WT was determined by DEseq2 based on *Q* ≤ 0.05 and fold-change ≥ 1.5. Color scale bar denotes the *z*-score transformed log_2_ ratios. **B)** GO enrichment of genes positively or negatively regulated by SA. Only GO terms with −log_10_FDR > 5 and fold-enrichment > 9 are shown. The RNA-seq experiment was conducted once.

We compared rosette transcriptomes of WT and *Fd-Irp9*-OE plants at bolting (stage 5.1; Boyes et al. 2001) under standard growth chamber conditions. The transcriptional response corresponded positively with SA levels, with 984, 890, 651, and 182 differentially expressed genes (DEGs, relative to WT) in F51, F36, F31, and F24, respectively, and none in F19 (Supplementary Data Set 1). We detected 1,825 DEGs in the SA-deficient *NahG* line. The 2 plants with the highest numbers of DEGs, F51 and *NahG*, shared 471 DEGs. Hierarchical clustering analysis of these 471 DEGs across all genotypes identified 2 main gene clusters with either SA-induced or SA-repressed expression (Fig. 3 and Supplementary Data Set 1). Genes positively regulated by SA were enriched for the gene ontology (GO) terms “systemic acquired resistance (SAR)” and “response to SA” and various defense and signaling pathways (Fig. 3, A and B), as would be predicted given the key function of SA in defense signaling. Examples of genes positively regulated by SA include the known SA markers *PATHOGENESIS-RELATED1* (*PR1*), *PR2*, and *PR5* (Uknes et al. 1992) and WRKY transcription factor genes implicated in SAR (Wang et al. 2006).

Interestingly, the transcriptional responses differed within the *NONEXPRESSOR OF PR1* (*NPR*) family encoding SA receptors. *NPR3* and *NPR4*, but not *NPR1*, were induced in hiSA lines (Fig. 3A), which presumably reflects their distinct roles in SA signaling (Ding et al. 2018; Tran et al. 2023). Expression of pattern-triggered immunity and effector-triggered immunity marker genes (Yuan et al. 2021) was unaffected in hiSA lines (Supplementary Data Set 1), except *FLS2* (*FLAGELLIN-SENSITIVE2*) and *CERK1* (*CHITIN ELICITOR RECEPTOR KINASE1*) which were significantly changed only in the extreme (*NahG*, F36, and F51) genotypes (Fig. 3A and Supplementary Data Set 1). Also unaffected was the expression of endogenous SA biosynthesis genes or their upstream regulators (Peng et al. 2021) (Supplementary Data Set 1), but *SARD1* (*SAR DEFICIENT1*) was upregulated by SA (Fig. 3A). *S3H* and *S5H*, which encode SA 3-hydroxylase and SA 5-hydroxylase for SA catabolism into 2,3-DHBA (2,3-dihydroxybenzoic acid) and 2,5-DHBA, respectively (Fig. 1A) (Zhang et al. 2013, 2017), were upregulated in an SA-dose-dependent manner (Fig. 3A). The results are consistent with increased accumulation of 2,3-DHBA and 2,5-DHBA conjugates in hiSA leaves (Fig. 1C).

The smaller cluster of genes negatively regulated by SA showed significant GO enrichment for “cold acclimation” and “response to water deprivation” (Fig. 3B). In particular, members of 4 *COLD-REGULATED* (*COR*) gene families [*COR15*, *COR6.6* (*KIN*), *COR47* (*RD17* and *ERD10*), *COR78* (*RD29*) and their tandem duplicates, except when below detection] known to be induced by cold (Thomashow 1999) were downregulated in the hiSA lines (Fig. 3A). *COR* genes are regulated by C-REPEAT/DEHYDRATION RESPONSIVE ELEMENT-BINDING FACTORS (CBFs/DREBs) and INDUCERS OF CBF EXPRESSION (ICEs) best characterized for their roles in freezing tolerance (Thomashow 1999; Kim et al. 2015; Tang et al. 2020; Li et al. 2024). *ICE* expression was low under nonstress conditions and unchanged in the hiSA lines. Among the *CBFs*, we detected only *CBF3* (*DREB1A*) transcripts, whose levels corresponded negatively with SA like *COR* genes (Fig. 3A), supporting sub-functionalization of CBF members (Novillo et al. 2007). Together, these results confirm canonical SA responses in hiSA leaves while also uncovering a negative effect of SA on *COR* gene expression.

### SA-mediated growth penalty is exacerbated at below-ambient temperatures

**Figure 4.**
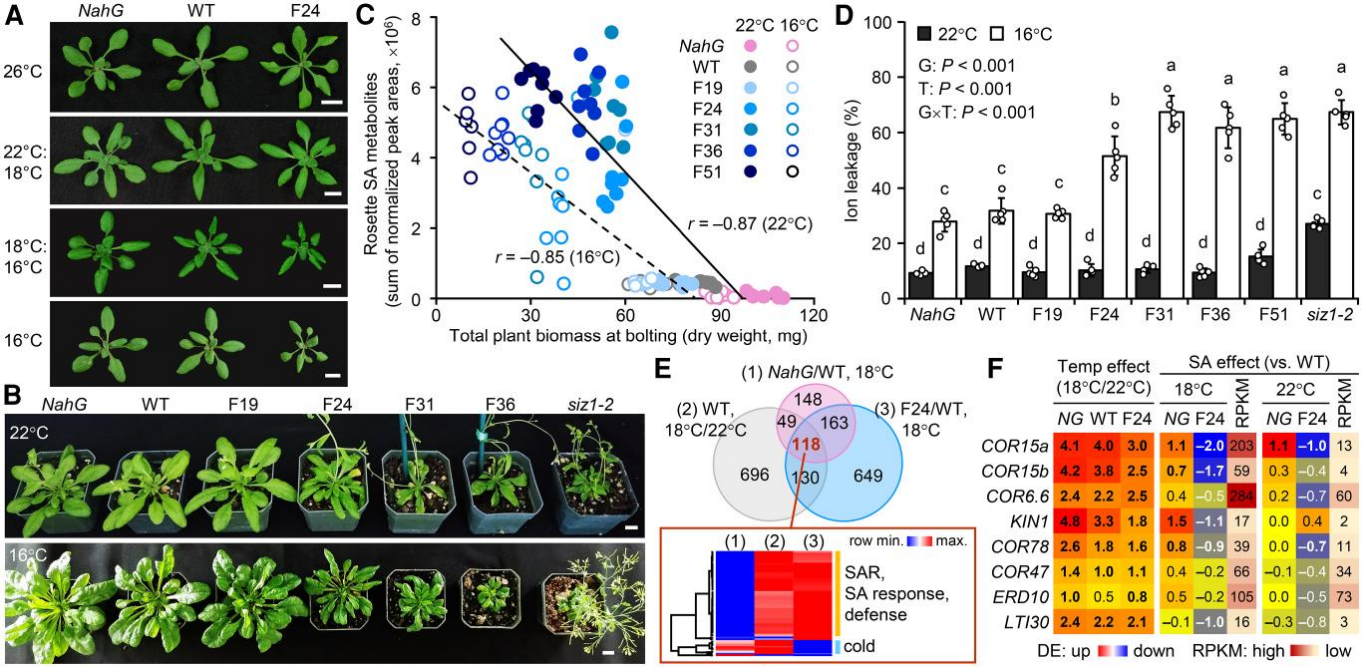
Temperature-sensitive growth and transcriptomic responses of the *Fd-Irp9*-OE lines. **A)** Representative phenotypes of *NahG*, WT, and F24 plants at 26 °C (18 DAG), 22 °C:18 °C (day:night, 18 DAG), 18 °C:16 °C (day:night, 26 DAG), or 16 °C (22 DAG). Scale bars, 1 cm. Each condition was repeated at least once with similar results. **B)** Representative phenotypes of *NahG*, WT, *Fd-Irp9-*OE (low to high SA, left to right), and *siz1-2* plants at 22 °C (30 DAG) or 16 °C (49 DAG). Scale bars, 1 cm. **C)** Regression analysis between total plant biomass and total rosette SA levels of multiple genotypes grown at 22 °C or 16 °C (*n* = 8 plants except for F51 at 16 °C where *n* = 7 plants). SA metabolite data are shown in Supplementary Figure S3A. **D)** Percent ion leakage of rosette leaf 8 from plants grown at 22 °C or 16 °C. Data are means ± SD (*n* = 5 plants). Effects of genotype (G), temperature (T), and their interaction were determined by 2-way ANOVA, followed by Tukey's post hoc tests; significance is indicated by letters. The experiments were conducted 4 times for growth monitoring, twice for SA measurements, and once for ion leakage analysis. **E)** Venn diagram of DEGs in response to SA (*NahG* vs. WT or F24 vs. WT) at 18 °C or to temperature changes (18 °C vs. 22 °C) in the WT. Clustering analysis of the intersection of 118 genes is shown below, and the top GO terms are indicated on the right. Color scale bar denotes the *z*-score transformed log_2_ ratios. **F)** Expression response heatmaps of *COR* genes. Values are log_2_-transformed ratios (significant difference denoted by boldface). Average transcript abundance (RPKM) of WT samples at 22 °C or 18 °C is also shown. Data were obtained from *n* = 4 independent pools, each sampled from 3 rosette leaves per plant at bolting. Differential expression between the specified sample pair was determined by DEseq2 based on *Q* ≤ 0.05 and fold-change ≥ 1.5. The RNA-Seq experiment was performed once. *NG*, *NahG*.

Motivated by the widespread downregulation of *COR* genes in the hiSA lines and by our anecdotal observations of greater growth penalties during winter when plants were watered with cold tap water, we conducted a series of temperature experiments to characterize the effects of SA on growth in more detail. We first compared F24 with WT and *NahG* plants under different temperature regimes (Fig. 4). Plant growth decreased as expected in all 3 genotypes as the temperature decreased from 26 °C to 16 °C, but the penalty was most pronounced in F24 (Fig. 4A). When we compared multiple hiSA lines at 22 °C and 16 °C, their growth reduction was exacerbated at below-ambient temperature in an SA-dose-dependent manner (Fig. 4, B and C, and Supplementary Fig. S3). Plants grown at 16 °C showed significantly greater ion leakage than those at 22 °C, and the differences corresponded positively with SA levels (Fig. 4D). Elevated ion leakage resulting from decreased membrane permeability is associated with chilling-induced injury and growth reduction (Lyons 1973). The data thus hint at a temperature-sensitive link between SA, membrane integrity, and plant growth.

We performed transcriptome analysis using rosette leaves of F24, WT, and *NahG* plants (stage 5.1) grown at 22 °C:18 °C (day:night, hereafter denoted as 22 °C) or 18 °C:16 °C (day:night, hereafter 18 °C) under otherwise identical long-day growth chamber conditions. We were particularly interested in genes responsive to SA perturbation at 18 °C (F24 vs. WT and NahG vs. WT) and to cool temperatures (18 °C vs. 22 °C) in the WT. The intersection of the 3 DEG lists (118 genes) showed 2 major groups (Fig. 4E and Supplementary Data Set 2). The first group consisted of 96 genes upregulated by both SA and cool temperatures, with GO enrichment for “SAR,” “response to SA,” and various defense responses (Fig. 4E). The second group (16 genes) was upregulated at below-ambient temperatures, but repressed by SA, and enriched for the GO term “response to cold.” Indeed, transcript levels of all leaf-expressed *COR* genes were significantly higher at 18 °C than at 22 °C in all three genotypes (Fig. 4F). However, the magnitude of *COR* gene induction was attenuated by SA, being lowest in F24 and highest in NahG (Fig. 4F). When compared with the WT, *COR* gene expression was significantly downregulated in F24 and upregulated in NahG at 18 °C (Fig. 4F). We observed a similar but weaker trend at 22 °C with overall lower *COR* transcript abundance (Fig. 4F). These results corroborated the findings (Fig. 3) of an inhibitory effect of SA on *COR* gene expression.

### Constitutive expression of COR genes rescues the growth defects of hiSA plants

COR proteins stabilize membranes during cellular dehydration, a common response to freezing and several other abiotic stresses (Thomashow 1999; Thalhammer et al. 2014). The suppression of *COR* genes in the hiSA lines might compromise this protective function at below-ambient temperatures. We therefore sought to test the hypothesis that SA-insensitive expression of *COR* genes can rescue the growth defects in the hiSA lines. We first attempted to overexpress *COR15a*, *COR15b*, or both (*COR15ab*) under control of the 35S promoter in WT and F24. All *Pro35S:COR15* transgenic plants in both backgrounds exhibited prolonged leaf expansion and delayed senescence (Supplementary Fig. S4, A–D), consistent with a promotive role of COR15a and COR15b in leaf longevity (Yang et al. 2011). While rosette and biomass growth improved at both 22 °C and 16 °C (Supplementary Fig. S4, E–G), total SA levels and disease resistance became attenuated in homozygous (T4) F24-*COR15* lines (Supplementary Fig. S4, H and I).

We attributed this flaw to 35S promoter-mediated cosuppression because both the original *Fd-Irp9* and the *COR15* constructs were built on pCambia vectors with double 35S promoters driving the selectable marker and the target genes (see Materials and methods). Shallow RNA-seq confirmed a potent silencing of the (abundantly expressed) *Fd-Irp9* to background levels in 2 F24-*COR15ab* lines, along with depleted transcript levels of *PR1* and other SA marker genes (Supplementary Fig. S4J). However, the relatively less expressed *COR15a* and *COR15b* were not silenced, indicative of dose-dependent differential cosuppression responses (Jorgensen et al. 1996). The results are consistent with the F24-*COR15ab* homozygotes reverting SA levels to WT-like but exhibiting prolonged leaf expansion characteristic of ectopic *COR* expression.

**Figure 5.**
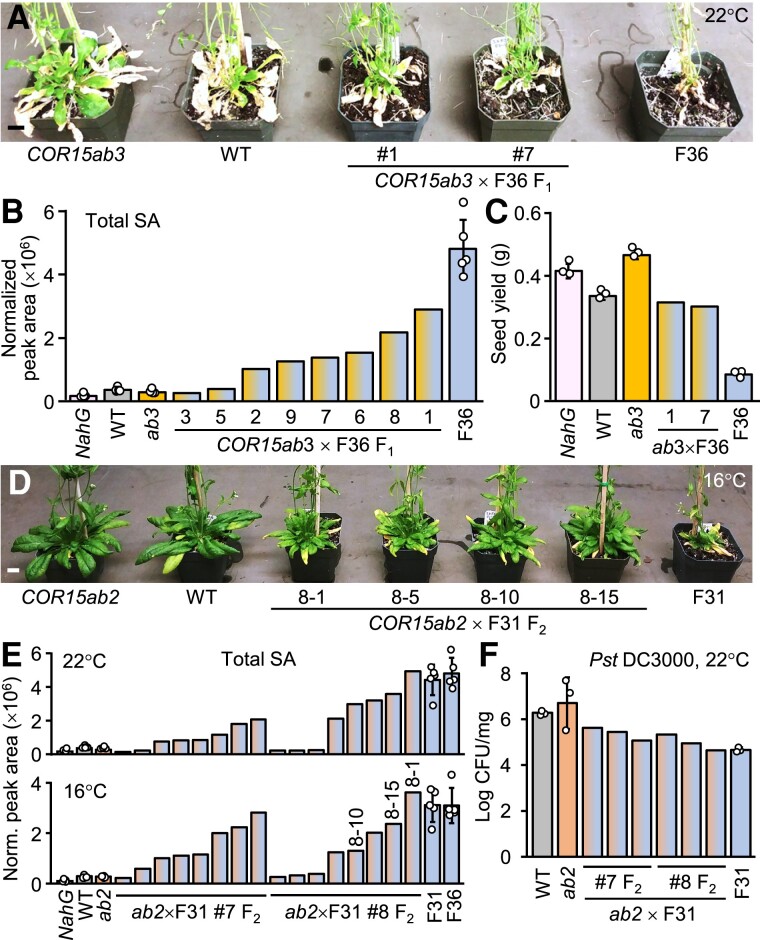
F_1_ and F_2_ progeny phenotypes of *Pro35S:COR15ab* and hiSA crosses before the onset of silencing. **A–C)** Plant growth at 65 DAG (A), total SA metabolite levels measured in leaf punches (B), and seed yield (C) of representative F_1_ plants from *COR15ab3* × F36 at 22 °C. Data for homozygous genotypes are means ± SD of *n* = 5 (B) or *n* = 3 (C) plants. **D–F)** Plant growth at 65 DAG at 16 °C (D), total SA levels in leaf punches at 22 °C or 16 °C (E), and *Pst* DC3000 disease resistance at 22 °C of representative F_2_ progeny from *COR15ab2* × F31 F_1_ #7 or #8. Data for homozygous genotypes are means ± SD of *n* = 5 (E) or *n* = 3 (F) plants. Each experiment was performed once. Scale bars, 1 cm.

In a second attempt, we examined crosses between *COR15ab* (WT background) and hiSA lines in early generations to obtain proof-of-concept data before the cosuppression effect intensifies in homozygous progeny (Fig. 5). In multiple crosses involving independent transgenic lines, F_1_ plant growth, leaf senescence, seed yield, and SA metabolite levels were intermediate of their parents at 22 °C (Fig. 5, A–C). We identified F_2_ individuals which maintained high levels of SA accrual and disease resistance like their hiSA parent, but with vastly improved growth at 16 °C (Fig. 5, D–F). This supports the hypothesis that severing *COR* suppression by SA could alleviate the growth penalty in hiSA lines.

**Figure 6.**
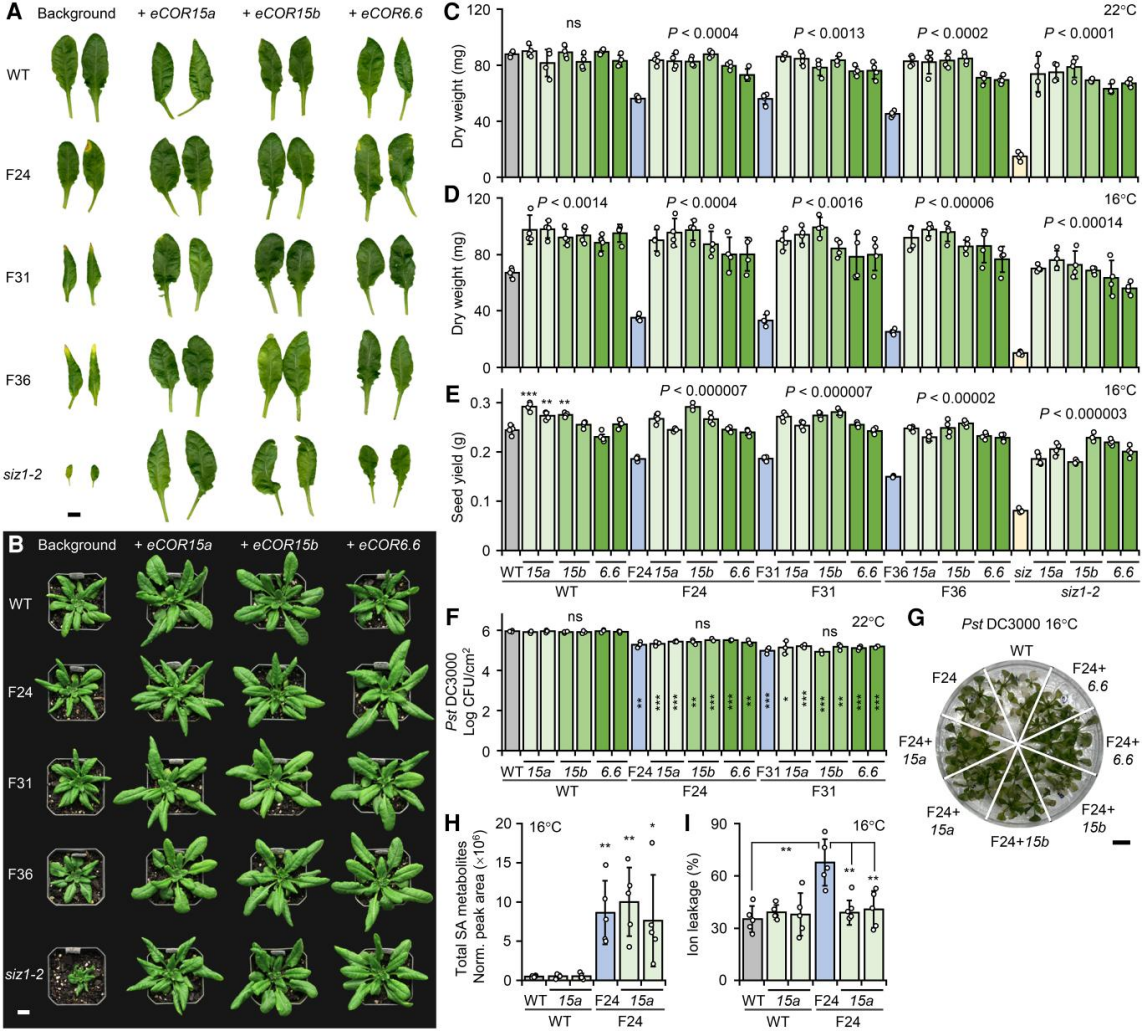
Constitutive *ProACT2:COR* expression rescues growth of SA-hyperaccumulating lines. **A)** Mature leaves of representative transgenic plants expressing *eCOR15a*, *eCOR15b*, or *eCOR6.6* in WT, *siz1-2*, and hiSA backgrounds at 22 °C (30 DAG). Scale bar, 1 cm. Whole plant images are shown in Supplementary Figure S6A. **B)** Plant growth of representative *eCOR* transgenic lines (49 DAG) at 16 °C. Scale bar, 1 cm. **C–E)** Rosette biomass (C and D) and seed yield (E) at 22 °C (C) or 16 °C (D–E) from 2 events per transformation (abbreviated without “*eCOR*”). Data are means ± SD of *n* = 4 plants. Statistical significance against the respective background was determined by 2-sided Student's *t*-test (***P < 0.001; **P < 0.01). **F)** Disease resistance against *Pst* DC3000 based on leaf infiltration of soil-grown plants at 22 °C (30 DAG). Data are means ± SD of *n* = 3 independent pools, each sampled from 2 rosette leaves per plant per pool. No statistical significance (ns) was found against the respective background based on 2-sided Student's *t*-test. Statistical differences against WT are indicated by asterisks inside the bar (***P < 0.001; **P < 0.01; *P < 0.05). **G)** Responses to *Pst* DC3000 based on flood inoculation of tissue-cultured plants at 16 °C. Scale bar, 1 cm. Additional replicates are shown in Supplementary Fig. S6. **H** and **I)** Total SA (H) and percent ion leakage (I) in rosettes of tissue-cultured plants at 16 °C. Data are means ± SD (*n* = 5 plants). Statistical significance was determined by 2-sided Student's *t*-test against WT in (H) or as indicated (I) (**P < 0.01; *P < 0.05). The growth experiments were conducted 3 times with reproducible phenotypes. All other analyses were performed once.

Next, we remade the constructs for ectopic expression of individual *COR* genes under control of the *ACTIN2* (*ACT2*) promoter in the WT, F24, F31, F36, and *siz1-2* backgrounds, hereafter referred to as *eCORs* (ectopic *CORs*). We chose *COR15a*, *COR15b*, and *COR6.6*/*KIN2* for 2 reasons: they were most responsive to cooling in F24 (Fig. 4F) and their encoded proteins reside in different subcellular compartments with distinct-yet-overlapping stress responses (Kurkela and Borg-Franck 1992; Wilhelm and Thomashow 1993). Consistent with the proof-of-concept data above, all 3 *eCOR* genes rescued hiSA and *siz1-2* plant growth to varying degrees (Fig. 6 and Supplementary Figs. S5 and S6). Due to prior experience of unexpected gene silencing, we prioritized our transgenic characterization on growth and SA phenotypes. We measured SA levels from one randomly selected plant per genotype from T_2_ plants to verify no apparent loss of SA accrual in hiSA-*eCOR* lines (Supplementary Fig. S6). We grew two randomly selected homozygous lines from each background-*eCOR* combination for further characterization at 22 °C and 16 °C under long-day conditions. All *eCOR* transgenic lines in hiSA backgrounds showed prolonged leaf expansion, improved growth, and significantly higher biomass over their respective parental lines at both temperatures (Fig. 6, A–D, and Supplementary Figs. S5 and S6). Specifically at 16 °C, hiSA plants expressing *eCORs* exhibited reduced growth penalty relative to the WT or WT-*eCOR* plants (Fig. 6, B and D). All *eCOR* lines showed delayed senescence as previously reported (Yang et al. 2011), which resulted in significantly higher seed yields than their cognate background at 16 °C (Fig. 6E) as observed for representative F_1_ hybrids of *Pro35S:COR15ab* and hiSA lines (Fig. 5C).

Across all backgrounds and at both temperatures, *eCOR15a* and *eCOR15b* appeared more effective than *eCOR6.6* in rescuing hiSA plant growth (Fig. 6, A–E). *eCOR* expression also significantly improved *siz1-2* growth at both temperatures but in many cases the biomass and seed yields were still significantly lower than those of WT or WT-*eCOR* lines (Fig. 6, A–E), suggesting partial rescue. This along with the observation that SA levels were partially depleted in the *siz1-2* background (Supplementary Fig. S6) again attests to SA accumulation as a pleiotropic phenotype of *siz1-2* (Park et al. 2011; Kim et al. 2021).

Ectopic expression of *COR* genes did not interfere with resistance to *Pst* DC3000 in the F24 and F31 backgrounds (Fig. 6, F and G, and Supplementary Fig. S6), consistent with sustained SA levels in hiSA-*eCOR* lines at either temperature (Fig. 6H and Supplementary Fig. S6). Furthermore, ion leakage was significantly lower in the F24-*eCOR15a* lines than in the F24 parent and equivalent to that of the WT or WT-*eCOR15a* lines at 16 °C (Fig. 6I). Together, the data suggest that it is possible to rescue hiSA plant growth by ectopic *COR* expression to restore membrane protection without compromising SA-mediated disease resistance.

## Discussion

**Figure 7.**
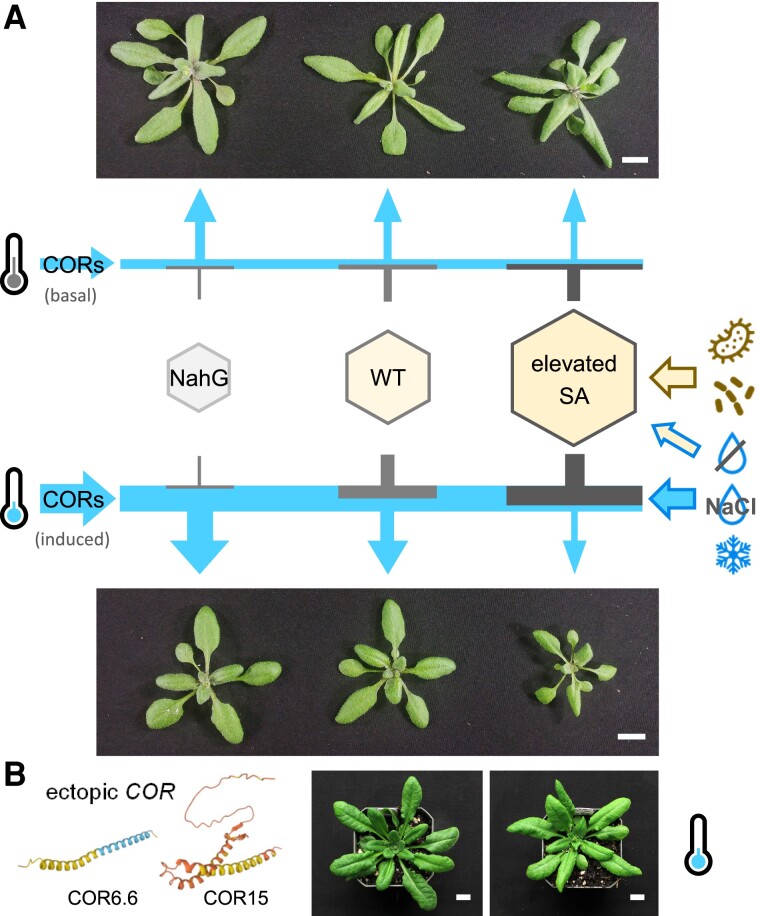
A proposed model for the growth–defense trade-off involving SA and CORs in response to various stressors. **A)** Plant growth under ambient conditions with basal or low levels of *COR* expression (blue arrows) is negatively affected by SA (top). CORs are strongly stimulated for membrane protection at below-ambient temperatures. This induction is attenuated by SA (gray flatheads), resulting in exacerbated growth reduction in hiSA plants (bottom). The negative regulation of *CORs* by SA may also affect growth–defense trade-offs in response to various biotic and abiotic stressors denoted by brown and blue arrows, respectively, on the right. **B)** Ectopic expression of individual *COR* genes can bypass SA suppression and rescue plant growth. Scale bars, 1 cm. The COR6.6 and COR15a structural predictions were retrieved from AlphaFold DB (https://alphafold.ebi.ac.uk).

The transgenic hiSA Arabidopsis lines reported here permitted unambiguous determination of SA effects on plant growth and stress responses. We show that elevated SA levels reduced biomass accrual, promoted precocious flowering and senescence, decreased seed yield, improved disease resistance, and enhanced abiotic stress tolerance, all in a dose-dependent manner. Notably, the hiSA lines exhibited more pronounced growth decreases at sub-ambient temperatures. Our data support a model in which SA antagonizes *COR* gene expression to fine-tune growth and defense responses to external cues (Fig. 7). The SA inhibitory effects appear modest in unstressed plants with basal or low levels of *COR* expression (Figs. 4F and 7A, top). With decreasing temperatures, *COR* genes are strongly stimulated but this induction is attenuated in hiSA plants (Fig. 4F), which exacerbates the growth reduction (Fig. 7A, bottom). A similar mechanism may also underlie plant responses to a range of biotic and abiotic stresses that activate SA and/or *CORs* to varying extents (Fig. 7A, right). We show that the SA inhibition of growth can be fully or partially rescued by transcriptional rewiring of individual *COR* genes to balance growth and defense (Fig. 7B).

COR proteins were originally identified by their rapid and strong induction in cold-acclimated Arabidopsis (Gilmour et al. 1988; Hajela et al. 1990; Kurkela and Franck 1990). Several of them were independently discovered in other screens with alternative names, such as cold-inducible (KIN), low temperature-induced (LTI), responsive to desiccation (RD), or early responsive to dehydration (i.e. COR6.6 = KIN2, COR47 = RD17, COR78 = LTI78 = RD29a) (Thomashow 1999). Perhaps less recognized is the sensitivity of *COR* genes to moderate cooling. Wang and Hua (2009) reported *COR* gene induction within hours of cooling (28 °C → 22 °C or 22 °C → 16 °C), though it was smaller, transient, and more variable than after cold shock (22 °C → 4 °C). Among our other findings in this report, we show significant and sustained induction across all *COR* gene families in plants grown at sub-ambient temperatures (Fig. 4F). The work adds to the known dehydration-related abiotic stresses that induce *COR* gene expression.

Cold temperatures reduce plant growth while increasing SA production and disease resistance (Scott et al. 2004; Carstens et al. 2014; Ibañez et al. 2017; Wu et al. 2019; Li et al. 2020). Moderate cooling also reduced plant growth but did not have large effects on SA accrual (similar levels at 22 °C and 16 °C; Supplementary Fig. S3) as previously reported (Li et al. 2020; Bruessow et al. 2021). Cooling stimulated *PR* gene expression in an SA-dependent manner, but SA is not required for cooling-induced *COR* expression (Wang and Hua 2009; Kim et al. 2013) (Fig. 4F). Instead, we show that SA had a detrimental effect on the induction of *COR* and other low-temperature-sensitive genes (Fig. 4F). This negative regulation of *COR* genes by SA has received little attention in the literature, but a majority (58) of the 100 SA-repressed genes we identified, including several *CORs* (Fig. 3A), was classified as downregulated by SA or SA-analogs in a meta-analysis (Zhang et al. 2020). Furthermore, an inverse relationship between basal SA levels and *COR* transcript abundances was gleaned from different Arabidopsis accessions where levels of *COR* genes were 1 to 2 orders of magnitude higher in Col-0 than C24 at midday (Miller et al. 2015). Diurnal expression of luciferase (*LUC*) under control of the *COR78* promoter was demonstrated in both accessions and the expression amplitudes were much higher in Col-0 than C24 for both *COR78(Col):LUC* and *COR78(C24):LUC* transgenes (Miller et al. 2015). The authors suggested that genetic backgrounds act *in trans* to modulate *COR78* expression (Miller et al. 2015). We argue that constitutively elevated SA in C24 (Bechtold et al. 2018) could underlie the suppression of *COR* genes relative to Col-0. Thus, multiple lines of evidence from the present and previous investigations support an inhibitory role of SA on *COR* expression in Arabidopsis.

SA and *CORs* have opposing roles in leaf senescence and longevity (Yang et al. 2011; Zhang et al. 2013, 2017). We posit that SA suppression of CORs likely contributed to the accelerated leaf senescence and flowering phenotypes in our hiSA plants (Fig. 1). This may represent an evolutionary strategy to optimize reproductive success in response to pathogen infection (Korves and Bergelson 2003). While SA has been implicated in both biotic and abiotic defense (Rivas-San Vicente and Plasencia 2011; Miura and Tada 2014; Peng et al. 2021), *COR* genes have mainly been associated with dehydration-related abiotic stress responses (Thomashow 1999; Miller et al. 2015). Our finding of SA-*COR* regulation thus links COR-mediated abiotic (including cooling) responses with SA defense signaling, and adds to the growing network of defense pathway interactions in response to diverse external and internal signals (Aerts et al. 2021; Liu et al. 2022).

Furthermore, the functional pleiotropy of CORs is linked to different regulatory circuits. Whereas COR induction by cold, drought, and other dehydration stresses is regulated by CBFs/DREBs and/or ICEs (Liu et al. 1998; Novillo et al. 2007; Kim et al. 2015; Tang et al. 2020; Li et al. 2024), and COR involvement in leaf longevity is regulated by NAC (NAM, ATAF1/2, and CUC2) transcription factor VNI2 (VND-INTERACTING 2) (Yang et al. 2011). Interestingly, *VNI2* expression was insensitive to SA or cooling in the present study, suggesting the involvement of other transcription factors in SA-COR regulation of leaf senescence and longevity. As discussed above, cold-activated disease resistance is SA dependent (Wu et al. 2019; Li et al. 2020). It has been shown that cold and pathogen defense signaling pathways share common players such as membrane receptors, calcium channels, reactive oxygen species, and mitogen-activated protein kinase cascades (Browse and Xin 2001; Ding et al. 2019; Wu et al. 2019). Specifically, ICE1 has recently been shown to physically interact with NPR1 and TGA3 (TGACG-BINDING FACTOR3) to activate SA signaling during cold-enhanced immunity (Li et al. 2024). However, expression of these genes was unaffected by SA or sub-ambient temperatures in the current study, although their involvement at post-transcriptional levels cannot be excluded. Whether the SA-COR regulation involves CBFs/DREBs, ICEs, or other transcription factors warrants further research. Given that leaf senescence is governed by an interplay of hormonal, developmental, and environmental cues (Jibran et al. 2013; Kim et al. 2017), the involvement of other defense–hormone crosstalk in the SA-COR regulation also requires further research.

All *COR* genes are present as tandem duplicates in Arabidopsis and closely related taxa that predate Brassicaceae speciation (Murat et al. 2015). As such, differences in basal expression (Fig. 4F) and stress responsiveness between paralogs have been reported. For instance, drought induces expression of *COR15a* and *COR6.6* (*KIN2*), but not *COR15b* or *KIN1* (Kurkela and Borg-Franck 1992; Wilhelm and Thomashow 1993). The *COR78* tandem duplicates (*RD29a* and *RD29b*) also show different basal expression and stress responsiveness (Yamaguchi-Shinozaki and Shinozaki 1993). At the protein level, COR proteins vary in size and subcellular localization (e.g. chloroplast for COR15 and cytosol for COR6.6), but they share characteristics of certain late embryogenesis abundant and dehydrin proteins, being highly hydrophilic and boiling stable (Gilmour et al. 1996; Kovacs et al. 2008; Thalhammer et al. 2014). Experimentally characterized COR15a and COR15b are unstructured in their fully hydrated state and form amphipathic α-helices upon dehydration to promote membrane association and stabilization (Thalhammer and Hincha 2014; Navarro-Retamal et al. 2016). Similarly, another SA-suppressed and cold-induced gene *LTI30* (Fig. 4F) encodes an intrinsically disordered dehydrin with Lys-rich segments that can fold into α-helices on the lamellar bilayer to stabilize membrane structures (Eriksson et al. 2011; Andersson et al. 2020). The other COR proteins are also predicted as intrinsically disordered hydrophilic proteins with amphipathic α-helical regions (Thomashow 1999), which suggests that they too may contribute to membrane protection.

Given the overlapping but nonidentical properties of *COR* genes and their encoded proteins and given the widespread suppression of *CORs* by SA, it may seem surprising that transcriptional rewiring of individual *COR* genes was sufficient to restore hiSA plant growth. Nevertheless, the results align with the notion that genetic redundancy underlies both evolvability and robustness of biological systems (Stelling et al. 2004; Kafri et al. 2009; Hunter 2022). The COR family exemplifies functional redundancy that can arise from duplicated genes (e.g. tandem duplicate) or distinct genes (e.g. different CORs) with overlapping function to buffer against stochastic perturbations, thereby increasing robustness and evolvability of the organism (Hartman et al. 2001; Stelling et al. 2004). *CORs* and other *LTIs* thus constitute a repertoire of “redundant genes” that can compensate for each other's loss (Kafri et al. 2009). In this context, our findings that any of the three *COR* genes tested could fully or partially rescue growth of hiSA mutants are not unexpected, after all.

There are precedents for successful uncoupling of growth–defense trade-offs mediated by another defense hormone, jasmonic acid (JA). JA inhibits growth by antagonizing gibberellin (GA) signaling in both dicot and monocot species (Yang et al. 2012; Heinrich et al. 2013). Accordingly, GA3 supplementation rescued growth defects of JA-activated wild tobacco (*Nicotiana attenuata*) caused by herbivory, MeJA treatment, or genetic lesions in the JA signaling pathway without affecting defense (Heinrich et al. 2013; Machado et al. 2017). In Arabidopsis, the growth penalty of a JA-hyperactivated mutant *jazQ* defective in 5 JAZ (jasmonate ZIM [ZINC FINGER INFLORESCENCE MERISTEM] domain) transcription repressors can be neutralized by another mutation in *PHYB* encoding the shade receptor phytochrome B (Campos et al. 2016). phyB suppresses both GA and growth-promoting phytochrome-interacting factors (PIFs), and its mutation in *jazQ* relieves the GA and PIF suppression in a manner similar to the shade avoidance response to promote growth (Campos et al. 2016). Thus, select de-repression with JA, GA, and light signaling networks via genetic or pharmacological means can alter growth–defense trade-off outcomes in ways that allow plants to maintain heightened insect defense and robust growth (Heinrich et al. 2013; Campos et al. 2016).

These studies elegantly demonstrated that the growth–defense trade-off is not merely constrained by carbohydrate reserves or energetically costly production of defense compounds (Heinrich et al. 2013; Campos et al. 2016; Machado et al. 2017). Instead, they support the alternative view postulated by Kliebenstein (2016) that growth–defense trade-offs are driven by negative interactions among intersecting signal transduction pathways. In a striking analogy, we show that de-repression of *CORs* can overcome the growth–defense trade-off mediated by SA. While the underlying regulatory circuit remains to be illuminated, the negative regulation of *CORs* by SA exemplifies another “hardwired” transcriptional interaction (Campos et al. 2016) which we posit to restrict leaf longevity upon activation of SA defense signaling.

The collection of hiSA transgenic lines reported here greatly aided in the molecular dissection of the trade-off between growth and SA-mediated defense. SA, a plant defense elicitor, has motivated development of structural analogs for commercial applications, but yield penalties have dampened the prospects of SA-based crop protection in agriculture (Walters et al. 2013). The finding that overriding SA inhibition through constitutive *COR15a*, *COR15b*, or *COR6.6* expression was sufficient to restore plant growth while retaining SA-endowed disease resistance suggests that a minimalist strategy can be effective for genetic improvement of crop productivity. The discovery of genes coordinately and oppositely regulated by cold and SA means that additional molecular targets can be exploited, in a combinatorial and iterative fashion, to decouple growth–defense trade-offs in diverse crops under changing climate conditions. We anticipate that the hiSA transgenic lines will be valuable for mechanistic investigation of SA crosstalk with other phytohormone and defense pathways.

## Materials and methods

### Plant materials and growth conditions

*A. thaliana* Columbia-0 (Col-0) accession, *cpr5-2*, *dnd1*, *siz1-2*, and *srfr1-4* seeds were obtained from the Arabidopsis Biological Resource Center (ABRC, Columbus, OH, USA). Transgenic *NahG* seeds in the Col-0 background (Reuber et al. 1998) were a gift from Frederick Ausubel, Massachusetts General Hospital. Seeds were stratified for 3 days at 4 °C and sown onto 5 cm square plastic pots containing Miracle-Gro Moisture Control potting soil (Miracle-Gro Lawn Products, Inc., Marysville, OH, USA) supplemented with *Steinernema feltiae* (Nemasys, BASF Corp., Research Triangle Park, NC, USA). Unless otherwise noted, plants were grown in a Conviron chamber (Conviron Ltd., Winnipeg, Canada) at 22 °C with 65W T8 cool-white fluorescent bulbs under a 16 h light (100 *µ*mol m^−2^ s^−1^) and 8 h dark photoperiod. Temperature experiments of soil-grown plants were conducted using 2 growth chambers set at constant 26 °C, 22 °C, or 16 °C, or variable 22 °C:18 °C or 18 °C:16 °C day:night temperatures as indicated. Plants were grown under ambient (22 °C) conditions until 14 DAG before half of them were transferred to the cool temperature growth chamber. Transgenic plant selection in tissue culture was carried out in a walk-in growth room maintained at 22 °C outfitted with 60W FLAT PANEL VEG (FPV24-A) light-emitting diode (LED) lighting (Barron Lighting Group, Glendale, AZ, USA) at 16 h light (100 to 120 *µ*mol m^−2^ s^−1^) and 8 h darkness. Cool temperature experiments of tissue-cultured plants were conducted using a Percival growth chamber (CU36L4, Percival Scientific, IA, USA) set at a constant temperature of 16 °C under a 16 h light (SciWhite PetriClear lighting at 100 *μ*mol m^−2^ s^−1^) and 8 h dark photoperiod.

*Nicotiana benthamiana* seeds obtained from the National Tobacco Germplasm Collection were sown on soil as above and maintained in a walk-in growth room at 22 °C with 16 h lighting at 400 *µ*mol m^−2^ s^−1^ provided by AgroLED iSunlight T5 White LED lamps. Plants approximately 2-month-old were used for leaf infiltration.

### Generation of transgenic Arabidopsis lines

Transgenic lines were produced via floral dip transformation (Clough and Bent 1998) using *Agrobacterium tumefaciens* strain C58 (Koncz and Schell 1986) carrying different binary constructs. To generate the *Fd-Irp9-*OE lines (Supplementary Data Set 3), Col-0 was transformed with the binary plasmid pCAMBIA1302-*Pro35S:Fd-Irp9* (Xue et al. 2013). T_1_ plants were selected on half-strength Murashige and Skoog (1/2 MS) medium with 20 mg L^–1^ hygromycin and confirmed by PCR using primers for *Irp9* and *HPT* encoding hygromycin phosphotransferase (Supplementary Table S1). Confirmed T_1_ plants were transplanted to soil for LC–MS screening of SA levels (see below) and seeds from selected lines were harvested. T_2_ plants were again confirmed by PCR as above and by LC–MS analysis of SA metabolites, and T_3_ seeds were harvested for antibiotic screening of homozygous lines. T_3_ and T_4_ plants were again analyzed for SA levels by LC–MS. From the first pilot transformation trial, one hiSA event was recovered (F24). Then, a larger T_1_ population from a second transformation trial was screened to select a panel of lines that represent a wide range of SA increases for further characterization.

For preparation of *Pro35S:COR15* constructs, the coding sequences of *COR15a* (At2g42540, ABRC stock no. U12858) and *COR15b* (At2g42530, stock no. U10423) were PCR-amplified using primers containing vector homology (Supplementary Table S1). The cDNAs were cloned into *Spe*I and *Pml*I digested pCM (modified from pCAMBIA2301; Swamy et al. 2015) via Gibson assembly (NEBuilder HiFi DNA Assembly Cloning Kit, NEB, Ipswich, MA, USA) and sequence verified to generate *Pro35S:COR15a* and *Pro35S:COR15b* constructs. For the construct containing both *COR15a* and *COR15b* (*COR15ab* for short), the *COR15a* cassette was PCR-amplified using primers with vector homology (Supplementary Table S1) and cloned into *Pro35S:COR15b* predigested with *Eco*RI and *Bam*HI to produce *Pro35S:COR15a*-*Pro35S:COR15b* (*Pro35S:COR15ab*) which was sequence confirmed. Floral dip transformation of WT and F24 was performed as above and transgenic plants were selected by 50 mg L^–1^ kanamycin without (WT background) or with 20 mg L^–1^ hygromycin (F24 background). T_1_ plants obtained from antibiotic selection were confirmed by PCR using *NPTII* (neomycin phosphotransferase II) and transgenic *COR*-vector primers (Supplementary Table S1). T_3_ plants were again PCR-checked using *Irp9*, *HPT*, *NPTII*, and transgenic *COR*-vector primers and tested for disease resistance (see below). Homozygous T_4_ plants were grown at 22 °C and 16 °C for biomass, SA measurements, and disease resistance analysis (only the 22 °C group).

A second set of *COR15a* and *COR15b* vectors was similarly prepared using primers that introduced sequences encoding C-terminal HA-tag and Strep-tag II, respectively (Supplementary Table S1). A *COR6.6* (At5g15970)-*Flag* fragment was synthesized as gBlocks (Integrated DNA Technologies, IA, USA) and cloned into pCM as described above. To avoid transgene cosuppression, the *Pro35S:COR15a-HA*, *Pro35S:COR15b-StrepII*, and *Pro35S:COR6.6-Flag* cassettes were PCR-amplified and Gibson-assembled into a *Spe*I/*Pme*I-digested p201N backbone (Addgene #59175) in which the kanamycin selection marker gene was controlled by the potato (*Solanum tuberosum*) *Ubiquitin3* promoter and terminator (Jacobs et al. 2015). The 35S promoter was swapped for the Arabidopsis *ACT2* promoter amplified from clone pAtA2pt-Ppo (a gift from Peter Lafayette, University of Georgia, Athens, GA, USA) by Gibson assembly to generate *ProACT2*:*COR* constructs. All constructs were verified by sequencing.

Arabidopsis floral dip transformation was performed as above in WT, F24, F31, F36, and *siz1-2* backgrounds and transgenic plants were selected by 50 mg L^–1^ kanamycin with 20 mg L^–1^ hygromycin (in hiSA backgrounds) or without (WT and *siz1-2* backgrounds). T_1_ plants were further screened by PCR using *Irp9*, *HPT*, *NPTII*, and transgenic *COR*-vector primers (Supplementary Table S1). T_2_ plants were selected based on antibiotic resistance and increased leaf longevity characteristic of ectopic *COR* expression. Two randomly selected events per construct in each background were analyzed by LC–MS for SA levels and advanced through T_3_ to obtain homozygous plants. Homozygous plants were grown at 22 °C and 16 °C for growth characterization, and a subset of plants were used for SA measurements and disease resistance analysis (see below).

### Growth monitoring and biomass analysis

Col-0 WT, homozygous *Fd-Irp9-*OE lines, *NahG*, and several autoimmune mutants were monitored for various growth parameters shown in Fig. 1. Rosette leaf (nos. 7–8) thickness and rosette canopy diameter of plants grown at 22 °C (*n* = 5 plants) were measured at bolting stage 5.1 (28 DAG) according to Boyes et al. (2001) and the plants were destructively harvested for rosette and root biomass. The number of lateral roots (>1 cm) was counted and dry weight was obtained after oven drying at 55 °C. Additional plants (*n* = 5) were monitored for the onset of leaf senescence and bolting. Seed yield and silique traits (number and length) were measured at 50 DAG. Similar growth monitoring experiments were conducted more than 10 times, including as background genotypes for subsequent *Pro35S:COR* and *ProACT2:COR* transformants, with reproducible trends.

Growth comparison at 22 °C and 16 °C of WT, *NahG*, and *Fd-Irp9-OE* lines was similarly performed using *n* = 8 plants for data shown in Fig. 4C, or *n* = 4 plants for replicate data shown in Supplementary Fig. S3B. Similar growth monitoring experiments were performed at least four times, including as background genotypes for subsequent *Pro35S:COR* and *ProACT2:COR* experiments, with reproducible patterns. The comparison among WT, *NahG*, and F24 was conducted more than 10 times under various growth temperatures from 16 °C to 26 °C. Growth monitoring of *Pro35S:COR* or *ProACT2:COR* transformants (2 randomly selected events per group) was conducted using *n* = 3–4 homozygous plants, except when F_1_ and F_2_ progeny from selected crosses were used. The experiments were performed once for *Pro35S:COR* plants and at least 3 times for *ProACT2:COR* transformants with similar trends. Biomass data were measured only once as shown in Fig. 6 from 30 DAG plants at 22 °C and 40 DAG at 16 °C. Statistical significance between each transgenic group and WT or between each transgenic *COR* line and its cognate background was determined using 2-sided Student's *t*-test.

### RNA-seq analysis

Three fully expanded rosette leaves (no. 7–9) per plant were snap-frozen in liquid nitrogen. Plants at growth stage 5.1 (Boyes et al. 2001) were used, which corresponded to 30 DAG at 22 °C (Fig. 3 and Supplementary Data Set 1) and 28 DAG at 22 °C or 38 DAG at 18 °C (Fig. 4, E and F, and Supplementary Data Set 2) in 2 separate experiments. Approximately 50–100 *µ*L volume of liquid nitrogen-ground powder was used for RNA isolation with a Direct-zol RNA MiniPrep Kit (Zymo Research, Irvine, CA, USA) and PureLink Plant RNA Reagent (Invitrogen, Waltham, MA, USA). RNA library preparation and Illumina NextSeq 500 sequencing (single end, 75 cycles) were performed at the Georgia Genomics and Bioinformatics Core of the University of Georgia (Supplementary Data Set 3). Sequence data were preprocessed as described previously (Xue et al. 2015) and mapped to the *A. thaliana* TAIR v10 reference genome using STAR v2.5.3a (Dobin and Gingeras 2015). Transcript abundance was estimated by featureCounts v1.5.2 (Liao et al. 2014) for differential expression analysis by DESeq2 v1.22 with multiple testing corrections (Love et al. 2014). DEGs were determined by reads per kilobase of transcript per million mapped reads (RPKM) ≥ 3, *Q* ≤ 0.05, and fold-change ≥ 1.5 using *n* = 4 plants (individual pools of 3 rosette leaves per plant per pool), except 1 contaminated F51 sample (Fig. 3 and Supplementary Data Set 1) that was mis-clustered with *NahG* samples in principal component analysis and was excluded. Only nuclear protein-coding genes were reported. Log_2_-adjusted expression values were *z*-score transformed for hierarchical clustering analysis using Morpheus (https://software.broadinstitute.org/morpheus) with Pearson's correlation as the distance metric. GO enrichment was performed using ShinyGO v0.76.3 (Ge et al. 2020). Venn diagrams were drawn with DeepVenn (Hulsen 2022), and expression ratios were visualized with HeatMapper Plus (https://bar.utoronto.ca/ntools/cgi-bin/ntools_heatmapper_plus.cgi).

### Metabolite analysis

SA metabolites were measured by reverse-phase high-performance liquid chromatography–mass spectrometry as detailed previously (Xue et al. 2013) using plants at bolting (stage 5.1). For screening of early generation transformants, one fully expanded rosette leaf (no. 8) was sampled using a biopsy punch (2 mm diameter) directly into the extraction buffer. For analysis of homozygous transgenic lines, rosette leaves (nos. 7–9) were flash-frozen in liquid nitrogen, ground to a fine powder, and an aliquot freeze-dried. Four leaf discs or 5 mg of freeze-dried leaf powder per plant was extracted in 200 *µ*L of extraction buffer (1:1 methanol:chloroform, v v^−1^) containing ^13^C_6_-cinnamic acid, D_5_-benzoic acid, and resorcinol as internal standards. Metabolite identity was confirmed with authentic standards for SA, 2,3-DHBA, 2,5-DHBA (Sigma-Aldrich, St. Louis, MO, USA), and SAG (Toronto Research Chemicals, Toronto, ON, Canada), previously fraction-collected compounds for 2,5-DHBG and SGE (Xue et al. 2013), or through MS fragmentation match against published data or National Institute of Standards and Technology (NIST) library for 2,5-DHBX, 2,3-DHBG, and 2,3-DHBX (Dean and Delaney 2008; Bartsch et al. 2010; Xue et al. 2013). The glycosides and xylosides were further confirmed by LC–MS and tandem MS analysis of *N. benthamiana* extracts from leaves infiltrated with 1 mm SA, 2,3-DHBA, or 2,5-DHBA (Supplementary Fig. S2). Except for the T_1_ screening (*n* = 1) and when noted otherwise, SA metabolite analysis of *Fd-Irp9-*OE lines was performed with *n* = 7–12 (T_2_) or *n* = 5–8 (homozygous) plants. The experiment with homozygous hiSA lines at ambient temperatures was performed at least 4 times with reproducible trends. Experiments comparing 22 °C and 16 °C plants were conducted twice with similar patterns. SA metabolite analysis of *Pro35S:COR* or *ProACT2:COR* transformants was performed with *n* = 6 (*Pro35S:COR*) or *n* = 5 (*ProACT2:COR*) individual plants, except for the confirmation analysis of *ProACT2:COR* T_2_ lines (*n* = 1 plant per line) in all genetic backgrounds. Statistical differences were determined by 2-sided Student's *t*-test against WT samples.

### Pathogenicity assay

Plant resistance to *P. syringae* pv. tomato strain DC3000 (*Pst* DC3000) was assessed for soil-grown plants under the specified conditions using syringe infiltration of rosette leaves as described previously (Lovelace et al. 2018). Two days before inoculation, *Pst* DC3000 cultures were grown in King's B medium (Sigma-Aldrich, St. Louis, MO, USA) supplemented with 50 mg L^–1^ rifampicin. *Pst* DC3000 inoculum was prepared at a concentration of 1 × 10^6^ colony-forming units (CFUs) mL^–1^ (OD_600_ = 0.1, diluted 1:100) in 25 mm magnesium chloride. Rosette leaves 7 and 8 were inoculated with bacterial suspensions. Four leaf discs (2 discs per leaf) were collected from each infiltrated plant at 2 DPI and homogenized for 2 min in 500 *µ*L of 25 mm magnesium chloride in a SpeedMill PLUS Bead Mill homogenizer (Analytik Jena AG, Jena, Germany). Homogenized samples were serially diluted, and 10 *µ*L of each dilution was plated on King's B medium containing 50 mg L^–1^ rifampicin. Bacteria were counted after 2 days of incubation at 28 °C, and data are presented as log_10_ CFU cm^−2^ (Fig. 2A). The experiment was performed 6 times for *Fd-Irp9-*OE lines (*n* = 3 plants) at 22 °C with similar results, including as background genotypes for the *Pro35S:COR* or *ProACT2:COR* transformants. The experiment for the *Pro35S:COR* transformants (*COR15a*, *COR15b*, and *COR15ab*) was conducted once in T_3_ and once in T_4_ (*n* = 3 plants), showing gradual loss of disease resistance in the F24 background due to cosuppression (Supplementary Fig. S4I). The experiment for *ProACT2*:*COR* transformants (*COR15a*, *COR15b*, and *COR6.6*) in various backgrounds (*n* = 4 plants) was conducted once at 22 °C (Fig. 6F). Statistical significance was determined by 2-sided Student's *t*-test against WT or their respective background genotype as indicated.

Resistance to *Pst* DC3000 was also evaluated by flood inoculation assays as described previously (Ishiga et al. 2011) using in vitro-cultured plants. After surface sterilization and stratification for 48 h at 4 °C, seeds sown on half-strength MS medium with 0.3% (w v^−1^) gellan gum (PhytoTechnology Laboratories, Shawnee Mission, KS, USA) were incubated at 22 °C with a 16 h light (100–120 *µ*mol m^−2^ s^−1^) and 8 h darkness photoperiod. Seedlings at 4 DAG were transferred to pathogenicity assay Petri dishes each containing 5 seedlings from different genotypes. Two days before inoculation, *Pst* DC3000 culture was prepared as above in 25 mm magnesium chloride containing 0.025% (v v^−1^) Silwet L-77. Two-week-old Arabidopsis seedlings were flooded with 20 mL of inoculum for 3 min. After removal of excess bacteria, plates were sealed with Micropore surgical tape (3 m, St. Paul, MN, USA) and incubated under normal growth conditions. At 3 DPI, rosettes were surface-sterilized by plate flooding with 20 mL of 5% (v v^−1^) hydrogen peroxide for 3 min, followed by washing three times with sterile dH_2_O. After blotting dry on sterile paper towels, 3 leaves per plant were weighed and homogenized as above and serial dilutions plated. Bacteria were counted after 2 days of incubation at 28 °C, and data are presented as log_10_ CFU mg^−1^ fresh weight tissue. The experiment was performed in a randomized complete block design where each Petri plate of 5−7 genotypes (1 plant per genotype) was an experimental unit and 5 biological replicates per genotype were included (*n* = 5 plants). The experiment for *Fd-Irp9-*OE lines was conducted twice with similar patterns (Fig. 2B). Statistical significance was determined by 2-sided Student's *t*-test against WT samples. The seedling flood inoculation assays were conducted once for the F_2_ progeny of *Pro35S:COR15ab2* × F31 at 22 °C (Fig. 5F) and once for the F24-*eCOR* lines at 16 °C (Fig. 6G and Supplementary Fig. S6C). For the latter, tissue-cultured plants (7 genotypes per plate, 3 plants per genotype) were prepared as above and grown at 22 °C for 10 days before transferring to a 16 °C growth chamber. Bacterial inoculation was performed 11 days after (3-week-old plants) for visual documentation of disease responses (Fig. 6G and Supplementary Fig. S6C).

### Abiotic stress assays

Surface-sterilized *NahG*, Col-0, F19, F24, F36, and *siz1-2* seeds were germinated as described and 4-day-old seedlings were transferred to vertically placed square Petri dishes (Simport, Quebec, Canada) containing half-strength MS alone or with 100 mm NaCl or 250 mm mannitol. Primary root length was measured on 11-day-old seedlings. The experiment consisted of 5 replicate plates per treatment group, each plate containing 2 seedlings per genotype. The experiment was performed twice with similar results. For plant response to methyl viologen (or paraquat dichloride; Sigma, St. Louis, MO, USA), surface-sterilized and stratified seeds were plated on half-strength MS either alone or containing 5 *µ*M methyl viologen. The plates were incubated at 22 °C with 16 h light (100–120 *µ*mol m^−2^ s^−1^). Germination response was recorded 7 days after sowing. The experiment consisted of 5 replicate plates per group, each with 10 seeds per genotype. The experiment was performed twice with similar results. Statistical significance was determined by 2-sided Student's *t*-test against WT samples.

### Ion leakage assays

Four 2 mm leaf discs (rosette leaf 8) per plant (*n* = 5 plants) were collected into 15 mL Falcon tubes containing 5 mL of ddH_2_O. Samples were shaken at 60 rpm for 4–6 h at ambient temperature. Conductivity was measured using a conductivity meter (Traceable Products, Friendswood, TX, USA). The samples were boiled for 30 min and cooled to room temperature, and conductivity was measured again as total ion leakage. Conductivity values were corrected using ddH_2_O as a blank. Ion leakage for each genotype was estimated from initial conductivity as a percentage of total conductivity. The genotype, temperature, and genotype × temperature effects were determined by 2-way ANOVA, and significant effects were analyzed post hoc with Tukey's honestly significant difference test using JMP Pro 16 (SAS Institute, Cary, NC, USA). The experiment was conducted once. The effects of ectopic *COR15a* expression in WT or F24 background on ion leakage were similarly assessed for tissue-cultured plants grown at 16 °C, using six 2 mm leaf discs from rosette leaf 8 per plant (*n* = 5 plants). Statistical significance was determined by 2-sided Student's *t*-test against WT and, for F24-*eCOR* lines, against the F24 background as well. This experiment was performed once.

### Statistical analysis

Statistical analyses were performed as described in each figure legend. Statistical data are provided in Supplementary Data Set 3.

### Accession numbers

Accession numbers of the genes used in this study are: *ACT2* (AT3G18780), *COR6.6* (AT5G15970), *COR15a* (AT2G42540), *COR15b* (AT2G42530), *FD* (At1g60950), and *Irp9* (CAB46570). RNA-seq data are available from the National Center for Biotechnology Information Sequence Read Archive under accession numbers PRJNA939115 and PRJNA942941.

## Supplementary Material

koae210_Supplementary_Data

## Supplementary data

The following materials are available in the online version of this article.

**Supplementary Figure S1.** SA metabolite analysis of T_1_, T_2_, and T_3_*Fd-Irp9-*OE plants (supports Fig. 1).

**Supplementary Figure S2.** LC–MS chromatograms and tandem MS fragmentation patterns of SA-derived metabolites (supports Fig. 1).

**Supplementary Figure S3.** SA metabolite levels and growth of *Fd-Irp9-*OE plants at 22 °C or 16 °C (supports Fig. 4).

**Supplementary Figure S4.** Characterization of homozygous F24-*Pro35S:COR* transformants (supports Fig. 5).

**Supplementary Figure S5.** Growth phenotypes of additional *ProACT2:COR* transgenic lines (supports Fig. 6).

**Supplementary Figure S6.** Additional phenotypes of *ProACT2:COR* transformants (supports Fig. 6).

**Supplementary Table S1.** Primers used in this study.

**Supplementary Data Set 1.** DEGs in response to SA changes at 22 °C.

**Supplementary Data Set 2.** DEGs in response to SA and below-ambient temperatures.

**Supplementary Data Set 3.** Results of statistical analyses.
