# Supplementary material for: Altering cold-regulated gene expression decouples the salicylic acid–growth trade-off in Arabidopsis
Source: Plant Cell. 2024 Jul 26;36(10):4293–308. doi: 10.1093/plcell/koae210 (PMC11448890; doi:10.1093/plcell/koae210)
Supplement: koae210_Supplementary_Data [file koae210_supplementary_data.zip › TPC2024BR00440DR1_Supplemental_Material.pdf]

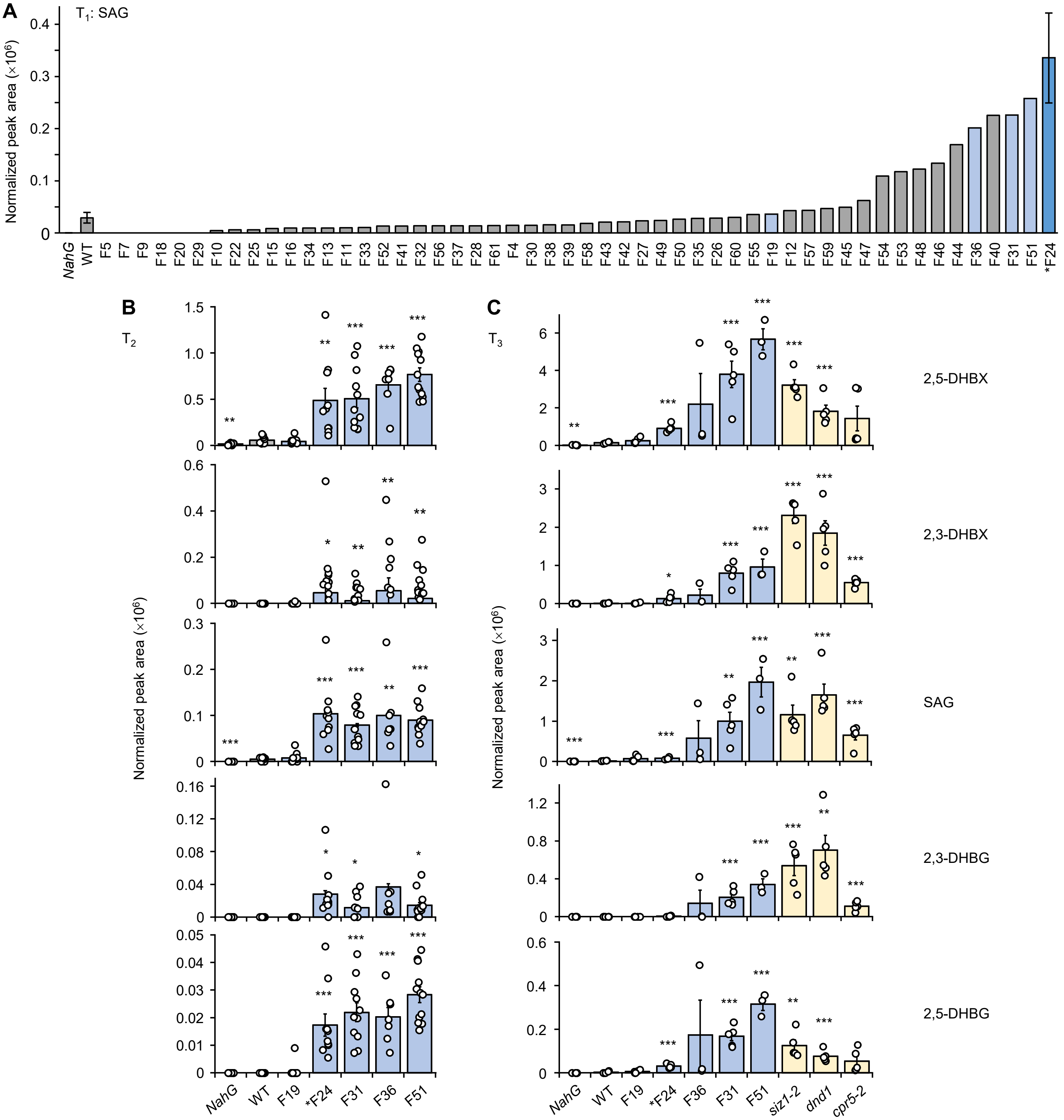

Supplementary Figure S1. SA metabolite analysis of T_1, T_2, and T_3 *Fd-Irp9*-OE plants (Supports Figure 1).
(**A**) Relative abundance of SAG in independent T_1 transgenic plants. WT, *NahG*, and homozygous (*) F24, the first high-SA line identified from a pilot-scale transformation, were included for comparison (means ± SD of n = 3 plants). Lines selected for further characterization are shown in blue. (**B**) Relative abundance of SA-derived metabolites in WT, *NahG*, and T_2 *Fd-Irp9*-OE lines (except *F24). Data are means ± SE (n = 7–12 plants). (**C**) Relative abundance of SA-derived metabolites in WT, *NahG*, T_3 *Fd-Irp9*-OE lines, and several autoimmune mutants. Data are means ± SE (n = 3–5 plants). Statistical significance was determined by two-sided Student's t-test against WT (***$P < 0.001$; **$P < 0.01$; *$P < 0.05$). All analyses were performed using four punches of a mature leaf at bolting. DHBG, dihydroxybenzoate glucoside; DHBX, dihydroxybenzoate xyloside; SAG, salicylic acid glucoside.

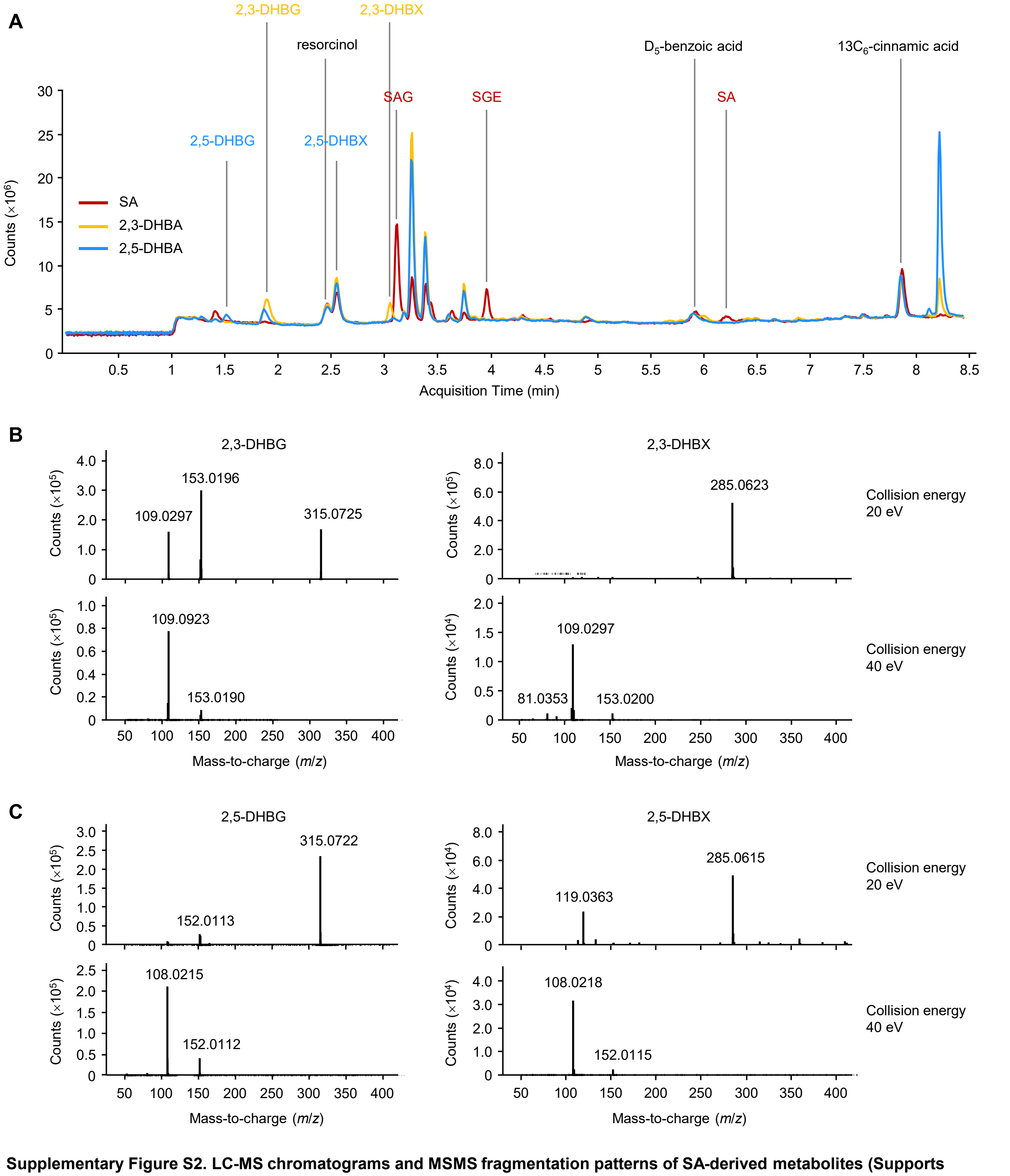

Supplementary Figure S2. LC-MS chromatograms and MSMS fragmentation patterns of SA-derived metabolites (Supports Figure 1).
(**A**) Chromatogram overlaps of tissue extracts from *Nicotiana benthamiana* leaves infiltrated with 1 mM SA, 2,3-DHBA, or 2,5-DHBA.
(**B-C**) Glycoside and xyloside conjugates identified from 2,3-DHBA (B) or 2,5-DHBA (C) infiltrated leaf extracts were analyzed by MSMS using collision energy at 20 eV and 40 eV. DHBG, dihydroxybenzoate glucoside; DHBX, dihydroxybenzoate xyloside; SA, salicylic acid; SAG, salicylic acid glucoside; SGE, salicylic acid glucose ester.

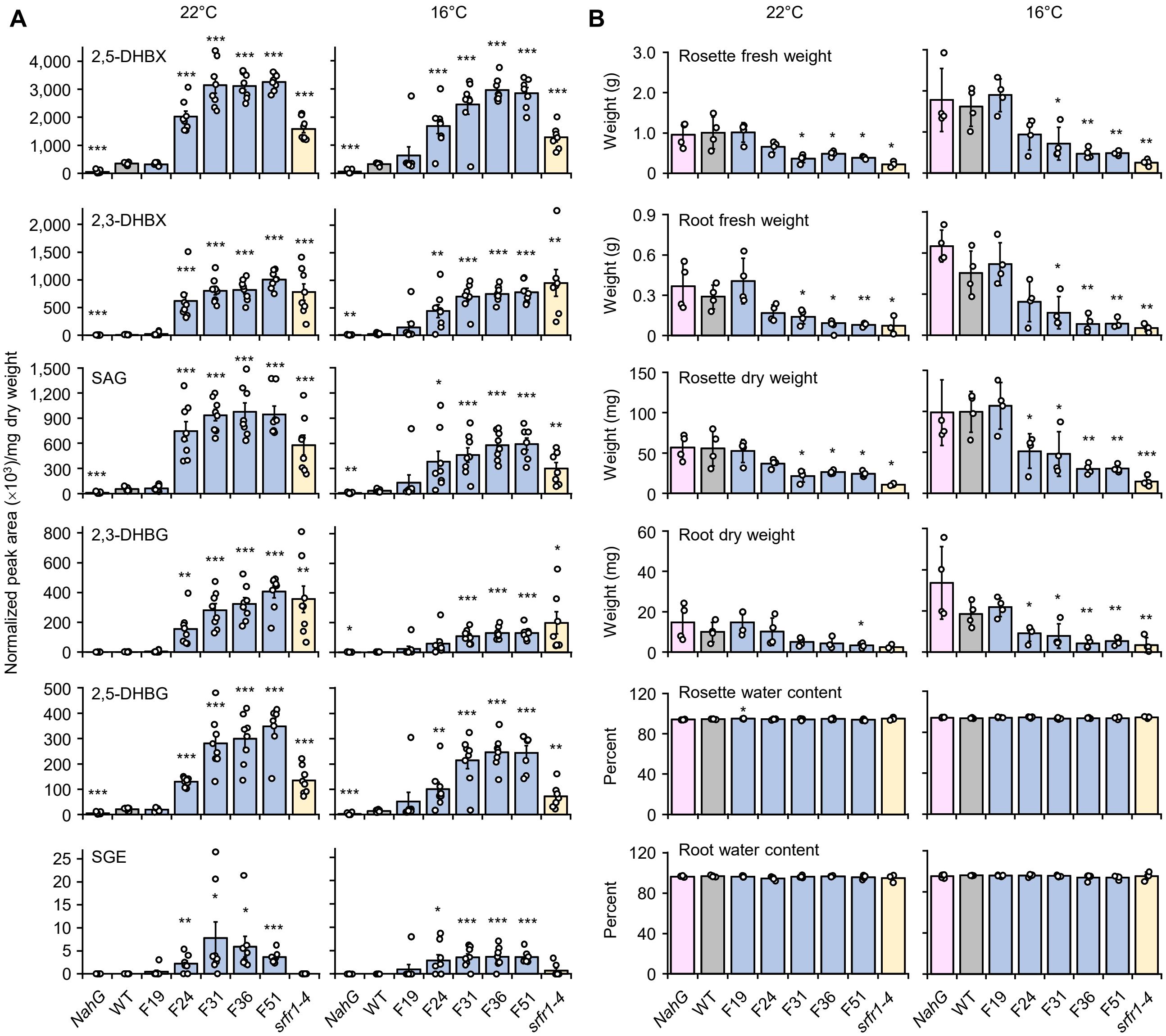

Supplementary Figure S3. SA metabolite levels and growth of *Fd-Irp9*-OE plants at 22°C or 16°C (Supports Figure 4).
(**A**) SA metabolite levels in mature rosette leaves (nos. 7–9) at bolting. Data are means ± SE of n = 8 plants. The sum of these metabolites was used for the regression analysis shown in Figure 4C. (**B**) Rosette and root biomass at bolting. Shown are fresh weight, dry weight, and water content from a replicate experiment in support of Figure 4B-C. Data are means ± SD of n = 4 plants, except for *srfr1-4* at 22°C (n = 3 plants). Statistical significance was determined by two-sided Student's t-test against WT (***P < 0.001; **P < 0.01; *P < 0.05). DHBG, dihydroxybenzoate glucoside; DHBX, dihydroxybenzoate xyloside; SAG, salicylic acid glucoside; SGE, salicylic acid glucose ester.

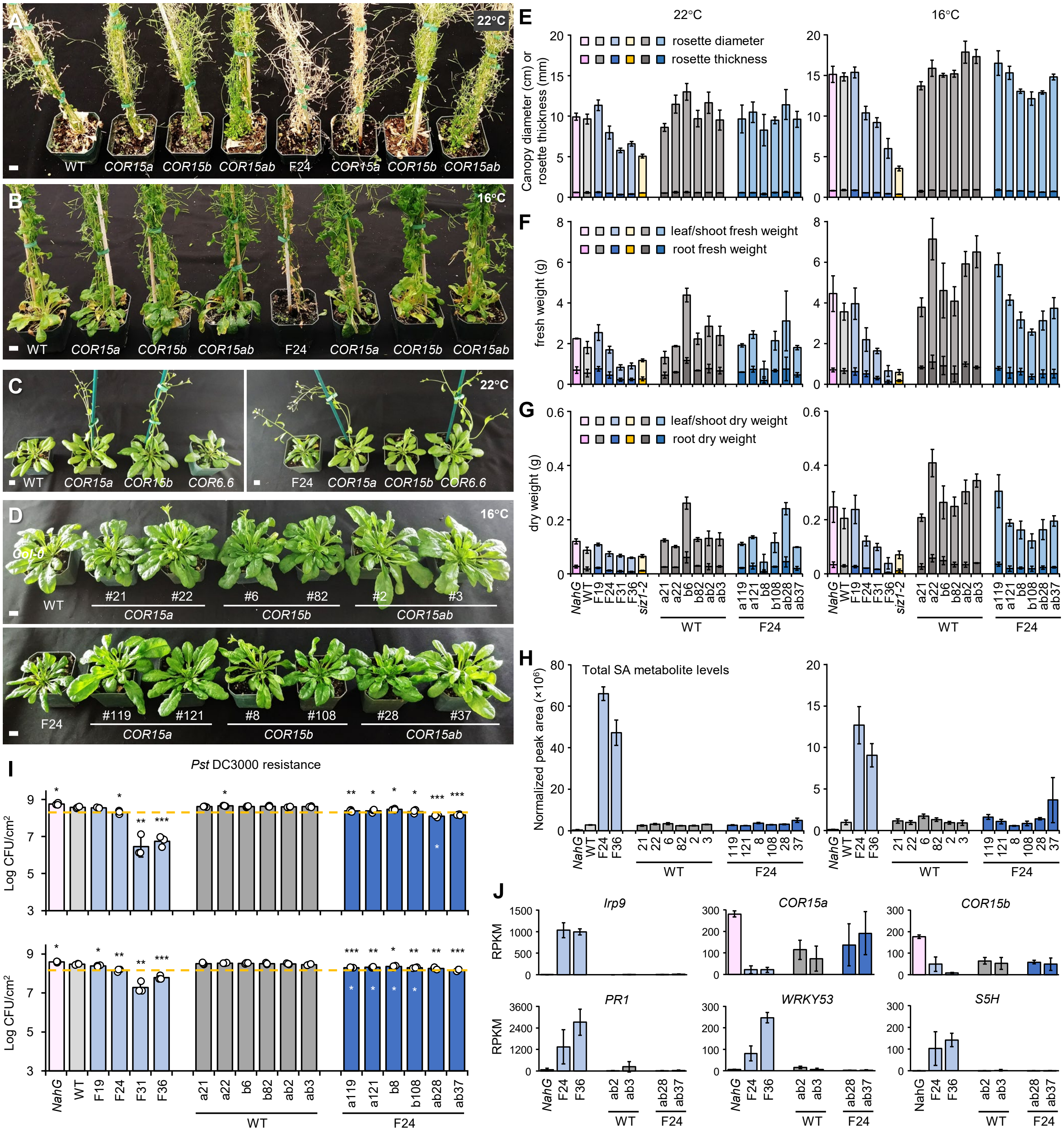

Supplementary Figure S4. Characterization of homozygous F24-*Pro35S:COR* transformants (Supports Figure 5).
(**A**-**D**) Representative T_3 (A, B) and T_4 (C, D) plants grown at 22°C (A, C) or 16°C (B, D) at 97 DAG (A-B) or bolting (C-D). Selected lines were used in crosses shown in Figure 5. Scale bars, 1 cm. (**E**-**G**) Canopy diameter, rosette thickness (E), fresh weight (F), and dry weight (G) of above-ground and root biomass. Data are means ± SD of n = 3 plants. (**H**) Total SA metabolite levels measured in rosette (no. 8) punches. Data are means ± SE of n = 6 plants at bolting. (**I**) *Pst* DC3000 bacterial growth based on leaf infiltration of soil-grown T_3 (top) and T_4 (bottom) plants at bolting at 22°C. Data are means ± SD of n = 3 plants. Statistical significance was determined by two-sided Student's *t*-test against WT (asterisks above the bar) or F24 (asterisks inside the bar). Yellow dotted lines denote F24 levels. ***P < 0.001; **P < 0.01; *P < 0.05. The experiment was performed once per generation. (**J**) Transcript levels of *Irp9*, *COR15a*, *COR15b*, and SA markers based on shallow RNA-seq. Data are means ± SD of n = 3 plants. WT RNA samples were compromised and not included.

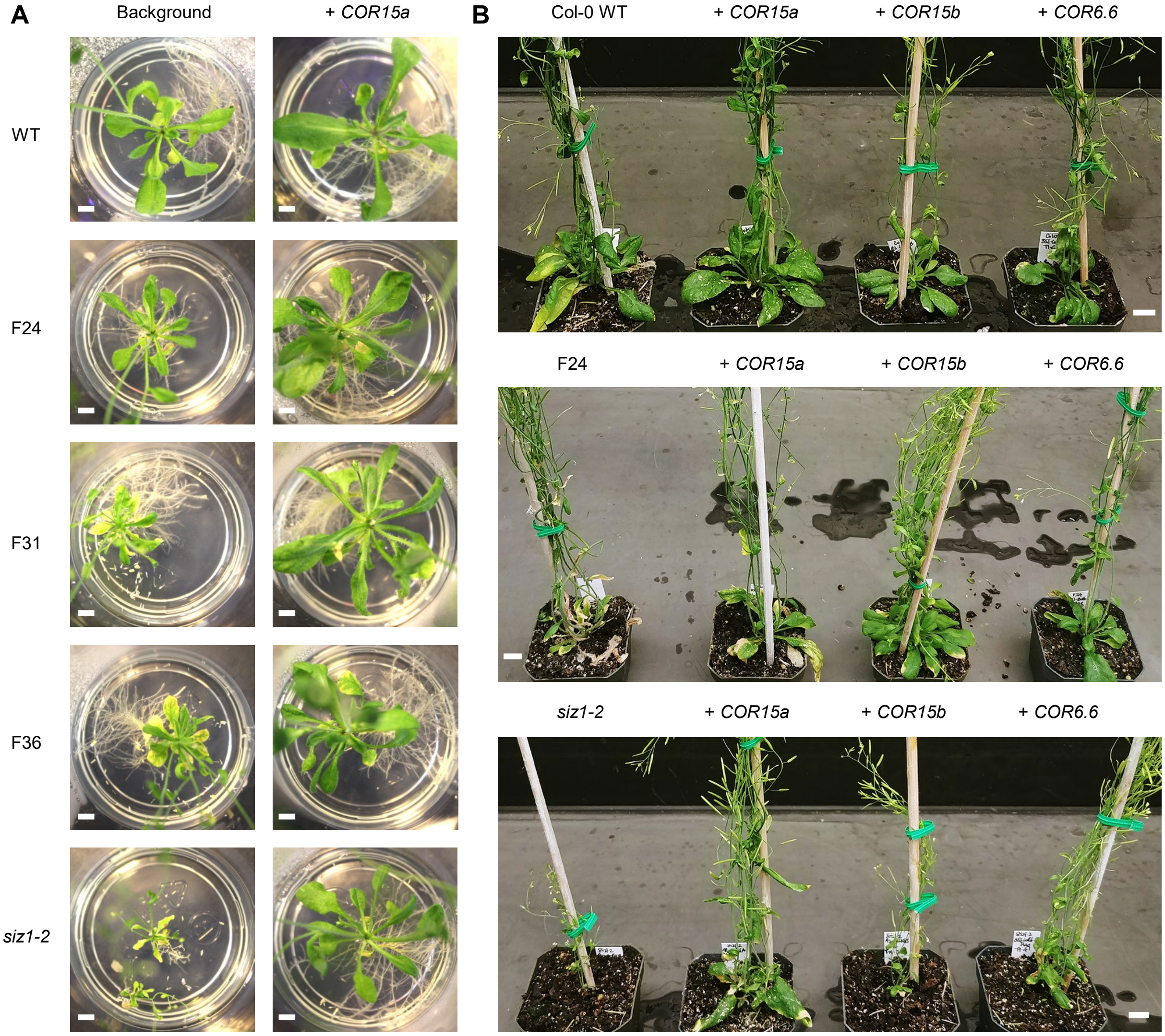

Supplementary Figure S5. Growth phenotypes of additional *ProACT2:COR* transgenic lines (Supports Figure 6). Additional transgenic lines not shown in Figure 6 grown on kanamycin and hygromycin selection media in tissue culture (**A**) or after soil transplanting at 22°C (**B**). Note the improved growth and delayed senescence relative to their cognate background. Scale bars, 1 cm.

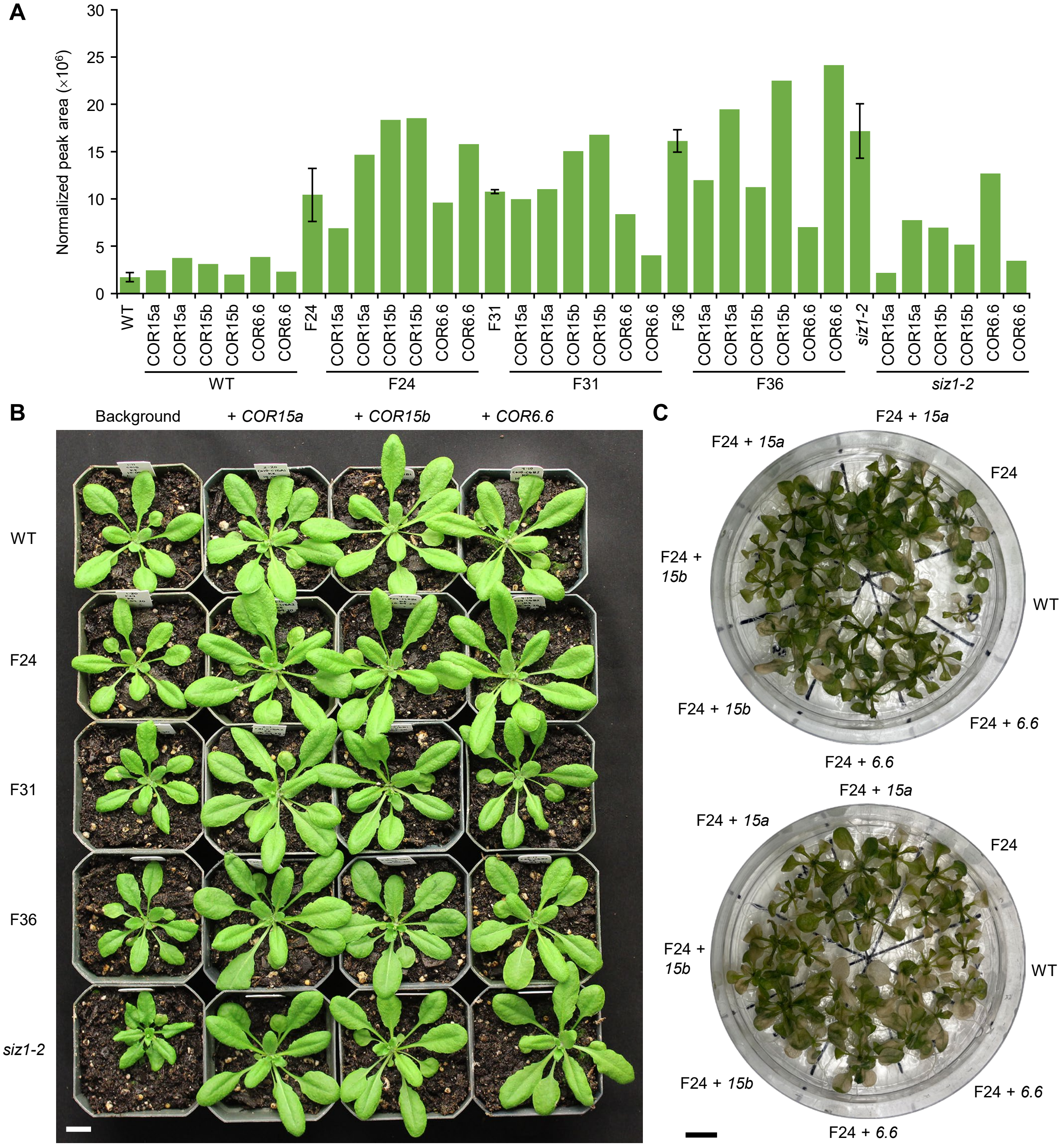

Supplementary Figure S6. Additional phenotypes of *ProACT2:COR* transformants (Supports Figure 6).
(**A**) Confirmation of total SA levels of T_2 plants grown at 22°C. One randomly selected plant per transgenic line or two plants per background genotype were analyzed using four punches of rosette leaf no. 8 at bolting. Data from the latter are means ± data range of n = 2 plants. (**B**) Whole plant image of representative transgenic plants ectopically expressing *COR15a, COR15b*, or *COR6.6* in WT, *siz1-2*, and hiSA backgrounds shown in Figure 6A (30 DAG, 22°C). Scale bar, 1 cm. (**C**) Additional replicates of 25 DAG *in vitro* seedlings at 16°C shown in Figure 6G. Photos were taken 4 days after *Pst* DC3000 flood-inoculation of WT and *COR* transgenic lines (two per construct) in F24 background. Scale bar, 1 cm.

Supplementary Table S1. Primers used in this study.

Primer	Sequence
Cloning of COR cDNAs into modified pCambia2301	
35SP.COR15A.F	AACACGGGGGACTCTTGACCATGGCGATGTCTTTCTCAGGA
PCM.COR15A.R	AATTCGAGCTGGTCACCAATCTACTTTGTGGCATCCTTAGCC
35S-COR15B.F	AACACGGGGGACTCTTGACCATGGCGATGTCTTTATCAGGAGC
pCM-COR15B.R	AATTCGAGCTGGTCACCAATACTATTGAACATGACTACATGAGTGG
pCM.EcoRI35S.F	cagctatgaccatgattacgaattCATGGAGTCAAAGATTCAAATAG
pCM.BamHINos.R	cctgcaggtcgactctagagAATACGgatCCGATCTAGTAACATAGATG
Cloning of COR-epitope cDNAs into p201N	
COR15A-HA.R	CTAAGCGTAGTCTGGCACATCGTAAGGGTACTTTGTGGCATCCTTAGCCT
HA(COR15A)NosT.R	AATTCGAGCTGGTCACCAATCTAAGCGTAGTCTGGCACATC
COR15B-SII.R	CTACTTTTCGAATTGTGGGTGAGACCAGGACTTTGTGGCATTCTTAGCCT
SII(COR15B)NosT.R	AATTCGAGCTGGTCACCAATCTACTTTTCGAATTGTGGGTGAGA
p201N rightborder.F	CTATCAGTGTTTGACAGGATATATTGGC
p201N stubi.R	TGGGCAGATCGATCCAAAGCACAT
p201N-speI 35S.F	GCTTTGGATCGATCTGCCCACTAGTGAGCATGGAGTCAAAGATTCAAAT
p201N-pmeI Nos.R	ATCCTGTCAAACACTGATAGAAACTGACCTGCAGGTCGACTCTAGAG
Replacing 35S promoter with AtACT2 promoter	
p201N-AtAct2P.F	TCGATCTGCCCACTAGTGAGTCATTATGTAAGAAAGTTTTGACGA
AtAct2P.COR15.R	AAGACATCGCCATGGTCAAGTTTATGAGCTGCAAACACACA
ACT2P-COR6.6F	GTGTGTTTGCAGCTCATAAACTTGACCATGTCAGAGACCAAC
p201N.Act2P.COR15.F	GTGTGTTTGCAGCTCATAAACTTGACCATGGCGATGTCTT
p201N.Act2P.R	CGTCAAAACTTTCTTACATAATGACTCACTAGTGGGCAGATCGA
Transgene genotyping	
Irp9(1013)F	ATGCGTTTACCGTGCTGTTTCCGT
Irp9(1291)R	AGGGCGCAATGCTCGCTAATTTCT
HPT1F	GAGGGCGAAGAATCTCGTGC
HPT2R	GATGTTGGCGACCTCGTATTGG
35SP.COR15A.F	AACACGGGGGACTCTTGACCATGGCGATGTCTTTCTCAGGA
NOST2	ATCGCAAGACCGGCAACAGG
35S-COR15B.F	AACACGGGGGACTCTTGACCATGGCGATGTCTTTATCAGGAGC
NOST2	ATCGCAAGACCGGCAACAGG
35SP1	CCCACTATCCTTCGCAAGACC
Flag-R	TCACCGTCATGGTCTTTGTAG
p201N.Act2P.COR15.F	GTGTGTTTGCAGCTCATAAACTTGACCATGGCGATGTCTT
COR15A-HA.R	CTAAGCGTAGTCTGGCACATCGTAAGGGTACTTTGTGGCATCCTTAGCCT
p201N.Act2P.COR15.F	GTGTGTTTGCAGCTCATAAACTTGACCATGGCGATGTCTT
COR15B-SII.R	CTACTTTTCGAATTGTGGGTGAGACCAGGACTTTGTGGCATTCTTAGCCT
ACT2P-COR6.6F	GTGTGTTTGCAGCTCATAAACTTGACCATGTCAGAGACCAAC
Flag-R	TCACCGTCATGGTCTTTGTAG
NPT1	GAACAAGATGGATTGCACGC
NPT2	GAAGAACTCGTCAAGAAGGC
AtNRX1(1360)F	GCTCTTCCATTTGGTGATCCTA
AtNRX1(1668)R	GTCCCTTCTTCCTCACACTTATC
NahG(1048)F	AACCTCGCCGAGCTGCTTGA
NahG(1241)R	AGGTCAGTGTCGAGGTCGTGGT
