# Supplementary material for: Altering cold-regulated gene expression decouples the salicylic acid–growth trade-off in Arabidopsis
Source: Plant Cell. 2024 Jul 26;36(10):4293–308. doi: 10.1093/plcell/koae210 (PMC11448890; doi:10.1093/plcell/koae210)
Supplement: koae210_Supplementary_Data [file tpc.00440.2024-s07.pdf › tpc.00440.2024-s07.pdf]

THE PLANT CELL

Decoupling the salicylic acid-growth tradeoff by altering cold-regulated gene expression in Arabidopsis

Maria Ortega, Rhodesia Celoy, Francisco Chacon, Yinan Yuan, Liang-Jiao Xue, Saurabh Pandey, MaKenzie Drowns, Brian Kvitko, and Chung-Jui Tsai

Review Timeline:

Submission Date:	09-Jun-2023
Editorial Decision:	08-Jul-2023
Resubmission Received:	14-May-2024
Editorial Decision:	07-Jun-2024
Revision Received:	10-Jun-2024
Accepted:	14-Jun-2024

Prof. Chung-Jui Tsai
University of Georgia
Athens, Georgia 30602

Dear Dr. Tsai:

Thank you for choosing to send your manuscript entitled "Decoupling the salicylic acid-growth tradeoff by altering cold-regulated gene expression in Arabidopsis" for consideration at The Plant Cell. Your submission has been evaluated by members of the editorial board as well as expert reviewers in your field, and we regret to inform you that we are not able to recommend the publication of this manuscript in its current form. We have had input from multiple scientists and we have solicited post-review comments.

During the post-review consultation session, we also agreed that if you could address the major points raised by the reviewers by new experiments, we would welcome a resubmission. We hope you can follow the reviewers' recommendations to improve the thoroughness of the study; the suggested experiments relate largely to missing controls, improved quantification, confirmation of observations, and better explanations of the results.

It will be important to convince the editors and reviewers that the study adds significant new understanding(s) of mechanisms or processes, or otherwise represents a significant advance in the field, and that the major claims made are fully justified by the data presented. This includes careful consideration and explanation of the various controls used in experiments, the extent and manner of replication, and the statistical analyses used. Sampling methods and the nature of "biological replicates" should be described precisely (i.e. different plants, parts of plants, pooled tissue, independent pools of tissue, sampled at different times, etc.), along with a clear description of and rationale for any statistical analyses conducted. The reader should know exactly what was sampled; what forms the basis of the calculation of any means and other statistical variables and parameters reported. This is also necessary to ensure that proper statistical analysis was conducted.

The Plant Cell now requires authors to complete and submit an author revisions checklist upon submission of a revised manuscript. The aim of the checklist is to aid authors in preparing a high-quality manuscript, facilitate the review and assessment of revised manuscripts, and help to ensure that journal standards are maintained across the board. If your manuscript is accepted, the completed checklist will be published as supplemental material attached to the article online. Please download a copy of the checklist (a fillable PDF form) at this link, for submission with your revised manuscript: https://tpc.msubmit.net/html/Author_Revisions_Checklist.pdf.

Note also that supplemental materials should be restricted to large datasets and tables, presentation of replicates, and validation of reagents, methods, or genotypes. Any data that are used to support the main claims must be in the main manuscript. Supplemental figure legends must indicate what figure in the main manuscript is supported by the supplemental data presented.

We thank you for your interest in and support of The Plant Cell. We hope we will be able to render a more positive decision on future work.

On behalf of the editorial board,

Reviewing Editor, Board of Reviewing Editors
Xin Li, Senior Editor
Blake Meyers, Editor-in-Chief

The Plant Cell

--- Reviewer comments:

Reviewer #1 (Comments for the Author):

This study by Ortega et al. utilized a bacterial SA synthase gene IRP9 to drive high level of SA in Arabidopsis to circumvent complex nature of defense response induction seen in autoimmune mutants. The resulting hiSA lines exhibited growth defects in a SA dosage dependent manner. The authors also identified a group of COR genes whose expression are inhibited by SA. Most strikingly, overexpressing three COR genes reversed the growth defects of the hi-SA plants.

It is well known that the plant defense hormone salicylic acid (SA) inhibits plant growth, but its underlying molecular mechanism is not fully understood. This study provides a possible link, that is COR gene expression, between SA and growth inhibition. It suggests that defense response and growth inhibition can be potentially uncoupled, which could lead to finer manipulation of defense responses without adversely affecting plant growth. This finding, when further substantiated, would be significant to the

field of plant-growth interaction.

The paper is well written. Experiments are well designed in general. Methods are explained in great detail. For instance, co-suppression was described although these lines were not reported. Data is in general of good quality.

Below are a few main concerns when addressed would substantiate the conclusion greatly.

1. Characterization of the hiSA COR-OE lines.
1) More lines need to be characterized. Now only two lines are shown although it is said that they were randomly selected. Additional lines could be looked at, but they do not need to be as thoroughly analyzed as the 2 existing lines.
2) Expression of IRP9 and COR genes should be analyzed in hiSA COR-OE lines and compared to hiSA lines and COR-OE lines. This is because hiSA COR-OE were not created by crossing of a hiSA line with a COR-OE line and therefore the expression may not be comparable.

2. Growth promoting phenotype of COR overexpression. Previous study did not find a growth promoting activity of COR genes. In fact, COR15 overexpression was reported to have slight growth inhibition (Thalhammer and Hincha, 2014). This discrepancy can be addressed. In particular, is this a 16C specific effect at least in wild type, as indicated by sFig 3b? COR genes are induced by a low temperature. Why would an overexpression of COR increase growth at 16C? Expression of COR genes can be analyzed in different COR-OE lines at both temperatures.

3. Quantification of growth defect.
The rosette size is not so different between hiSA lines (such as F24 and F31) and wt in picture (Fig. 4a), but the dry weight was 2-fold difference (Fig. 4b). Does that mean that fresh weight is not as different between the wt and the transgenic plants?
It would be good to quantify the fresh weights (which is the more commonly used in other growth-defense studies) so that the study can be more comparable to previous studies.
In addition, if hiSA COR-OE lines and the COR-OE lines differ in fresh weight (dry weight appeared to be same), it would be good to determine the interaction of effects from hiSA and from COR-OE to support the conclusion that COR-OE suppresses growth defect of hiSA rather than acting in an independent pathway of growth promotion.

4. SA-dependent growth defect as in L204. The exacerbated growth defect at 16C compared to 22C is stated as a SA dosage dependent. Although this is true among different lines at one temperature, SA accumulation is lower in 16C compared to in 22C while growth defect is more severe (Fig. S2). The exacerbated growth defect is therefore not from an increase of SA accumulation at 16C. This needs to be discussed. Is this more defense amplification or more inhibition of COR expression at low temperatures?

Additional points to consider:

1. Lines 63-74. Autoimmune mutants have various origins and there are many of them. It is good to cite a review or two to introduce autoimmune mutant comprehensively. SA accumulation is a usual feature, but activation of NLR genes is also common (Freh M, et al, 2022 Plant Physiol 188:1419). hiSA lines are not equivalent to autoimmune mutants where additional aspects of defense responses might be activated.

2. L188-192. Th genes listed, CPR5, DND1, SIZ1 and SRFR1, while their mutations lead to autoimmune, were not reported (or not relevant) to have their RNA expression changed upon SA induction in autoimmune mutants.

Reviewer #2 (Comments for the Author):

Dear Authors/Editor,

Ortega and colleagues have reported an exciting discovery of the molecular basis of Arabidopsis plant growth-defense trade-off mediated by the hormone salicylic acid. This is an important manuscript with implications in plant hormone signaling, abiotic stress tolerance, plant immunity and gene regulation. A major strength of this study is the successful implementation of the bacterial enzyme-based orthogonal approach (originally developed in poplar - Xue et al. 2013 Plant Cell; https://doi.org/10.1105/tpc.113.112839) in overproducing SA in Arabidopsis. This bypassed the pleiotropic effects of previously reported SA-overaccumulating mutants. In detail, the authors report that SA overaccumulation expectedly leads to growth defects in Arabidopsis, which is associated with upregulated in SA/SAR-related genes (as expected). Remarkably, they find that SA overaccumulation leads to downregulated expression of a suite of COR genes. The authors then "rescue" COR transcript levels by transgenic overexpression in their high SA mutants, which then boosts plant growth without sacrificing immunity to the hemibiotrophic pathogen P. syringae DC3000. In addition to this major conceptual advance, this study provides numerous plant materials (transgenic lines) and global transcriptome datasets to the research community. Finally, this exciting discovery could

pave the way for potential applications in bypassing growth-defense trade-offs in crop plants - a holy grail in the plant sciences.

Because of the broad implications of this study, the paper needs to properly show strong evidence to support the stated conclusions. It is also important to exercise caution that certain aspects of plant defences and growth still need to be investigated in future studies. Specifically:

1) The major thesis of this paper is that COR overexpression does not interfere with SA production and disease resistance in the high SA lines (F24 and F31) under different temperatures. However, there is no Pst DC3000 data in Figure 4 showing that disease resistance was maintained with COR overexpression at 16{degree sign}C. In parallel, there is no SA data in Figure S3 showing that hormone levels are unchanged after COR overexpression at 22{degree sign}C.

2) As a follow-up to #1, the authors overexpressed three different COR genes in their high SA mutant lines (as shown in Figure 4). Can the authors show confirmation of COR overexpression by providing COR gene expression data in their lines?

3) The paper shows that any of the COR genes tested could rescue the growth of high SA mutants without significant impacts on disease resistance. This is an intriguing finding that brings forth new questions. Why is it that a single COR gene is remarkably sufficient to uncouple this longstanding trade-off? The authors reason that their "results align with the notion that cellular robustness encompasses both functional redundancy and specificity" (Lines 265-266). Perhaps this can be more fleshed out in the Discussion section. Even speculation on potential mechanisms based on crosstalk between SA and growth hormones (BR, IAA, etc.) would be insightful to readers.

4) This is more philosophical but it would be great for the authors to highlight in the Discussion section why plants even evolved growth-defense trade-offs if simple COR overexpression is sufficient to uncouple this trade-off. Are there any hidden trade-offs of COR overexpression in plants? Are there natural instances (in certain Arabidopsis accessions) where the SA-growth trade-off is not apparent?

5) As a follow-up to #4, cold temperatures are known to induce both SA production and COR gene expression. So, why is it that growth suppression at this temperature is still observed? It may be good to discuss cold temperature regulation of plant growth that is SA-independent.

Minor comments:

1) Lines 60-61: It would be great to cite the seminal papers that postulated the growth-defense trade-off principle.

2) Lines 68-70: Apart from being an RNA-binding protein, CPR5 is also a component of the NPC (Gu et al., Cell; https://doi.org/10.1016/j.cell.2016.07.042). It would be great to at least acknowledge this.

3) Lines 63-64: Because the authors mention the dnd1 and srfr1 mutants in the first paragraph, it would be great to briefly provide their biological importance in this paragraph.

4) Lines 106-108: It is stated that Line F24 "had SA derivative levels that approached or surpassed those detected in the autoimmune mutants siz1-2 and dnd1." However, Figure 1b shows barely noticeable SAG/DHBX and SGE increases in F24. Based on this data, F24 resembles F19. I may have missed something, but can the authors please clarify this?

5) Lines 168-171: Apart from the NPR receptors and transcriptional coregulators, did the transcriptome also show upregulated expression of major SA-associated transcription factors, e.g. CAMTA1/2/3, SARD1 and CBP60g? How about PTI and ETI-related genes? Showing this data in Figure 2a may help broaden the paper's findings.

6) Lines 243-244: "Constitutive COR gene expression also rescued the growth defect of siz1-2 at both temperatures." - Based on the data in Figure 4b and Figure S3, I think this may be a partial rescue since the rosette dry weight does not approach that of the wild-type plants.

7) Line 250: I feel like this paragraph ends abruptly and could be missing a concluding sentence.

8) Lines 268-269: The statement on COR gene phylogeny needs a citation.

9) Line 323: Clarity the primers used to confirm the transgenics.

10) Line 355: Could the authors state the approximate number of times that the experiment was repeated with reproducible results?

11) Line 358, Lines 379-380 and Line 418: How old were the plants? Please clarify.

12) Lines 384-385: Indicate the supplies for the HPLC-MS standards used.

13) For Figures 1,3, 4 and S1 to S3: please clearly define n (Are they individual plants? Single pools of several plants? Individual leaves of the same plant?, etc.). In addition, please indicate the number of reproducible independent experimental repeats performed.

14) This may be a minor issue but it seems unconventional for the panel letters to not be chronologically arranged in Figures 1 and 3.

Response to review

Overview of major changes:
We wish to highlight two major changes, two clarifications, and key background info from the previous co-suppression experiments in response to reviewers' comments.

Major change 1: We have added substantially more data, including results from the (disappointing) co-suppression experiments. As explained below, those data were key in guiding our subsequent research efforts prioritizing trait over expression characterization.

Major change 2: We have overhauled the discussion to provide a more thorough synthesis of our findings against the backdrop of multiple intersecting effects on SA-COR regulation on plant growth, leaf expansion/senescence, biotic defense, and abiotic responses.

Clarification 1: We opted for re-transformation of hiSA lines rather than making crosses because of funding and time consideration; one must first generate homozygous *COR* transgenics and perform sufficient analyses to select events that do not display off-types before crossing. Even for Arabidopsis, this is a lengthy process. The re-transformation strategy allowed us to directly test our hypothesis about CORs more quickly. Time was even more pressing in our second attempt with *ACT2:COR* constructs, after ~2 years with the *35S:COR* transgenics, not to mention the ensuing global pandemic challenges. We would like to note that re-transformation is a well-established method for many species, especially those that have longer generation times or when crossing is not possible.

Clarification 2: While CORs have well-established function in cold tolerance, this study revealed their less acknowledged roles in leaf expansion and senescence at both ambient and subambient temperatures. It is the latter that are antagonized by SA, a pattern that is also supported by other published studies.

The cosuppression experiments: The *35S:COR* transformants were originally double selected by hygromycin (*35S:Fd-Irp9*) and kanamycin (*35S:COR*) in tissue culture and by PCR confirmation using Irp9, HPT, NPTII, and COR+vector primers through the T_3 generation to identify homozygous lines. These plants have very distinct phenotypes of prolonged leaf expansion and delayed senescence in both WT and F24 background, consistent with a previous report (Yang et al., 2011) and as shown in the new Fig. S5 (also further detailed in our response to review comments below). These phenotypes were already noticeable in early generations and were especially striking in the hiSA backgrounds which otherwise displayed premature senescence.

We tested *Pst* DC3000 disease resistance and found no significant differences between T_3 lines and their respective (WT or F24) background (new Fig. S4I, top panel). Unfortunately, our LC-MS was down during that time so SA levels were not measured until T_4. By then, we had lost SA accrual in the homozygotes (now Fig. S4H). We noted that *Pst* DC3000 resistance became weaker in T_4, as bacterial growth in F24-*COR* lines was significantly higher than F24, but lower than WT (Fig. S4I, bottom panel). In retrospect, the differences in T_3 were small but the statistical significance misled us until we obtained the SA data from T_4 plants. With the realization of potential cosuppression, we performed shallow RNA-seq to confirm that *Irp9* expression was indeed silenced in T_4 F24-*COR* lines, from RPKM ~1000 (in F24) to <10 (Fig. S4J). Interestingly though, neither *COR15a* nor *COR15b* was silenced as similar transcript levels were detected in WT-*COR* (ab2 and ab3) and F24-*COR* (ab28 and ab37) lines. Thus, cosuppression disproportionally affected the highly abundant *Irp9* transcripts (ranked among top 100 in F24 and F36) relative to the less expressed *COR15*s. Detection of *COR15* expression is consistent with prolonged leaf expansion and delayed senescence that we consistently observed, even in *Irp9*-silenced T_4.

We provide this background information here for two key takeaways and hard lessons. First, there are clear COR phenotypes in all selected transgenic events of WT and F24 backgrounds (12 independent lines total) throughout all generations we monitored. Second, SA levels are the most critical trait for our investigation. Gene expression has not been a reliable indicator based on COR phenotypes and shallow RNA-seq. We interpret the COR phenotypes as driven by constitutive/ectopic expression rather than overexpression *per se*. While we re-made the constructs to repeat plant transformation, we performed crosses between hiSA lines and COR15ab2 or COR15ab3 (WT background). We obtained proof-of-concept data that it was possible to obtain F_1 and F_2 hybrids with both high SA/disease resistance (hiSA phenotypes) and improved growth (COR phenotypes). These have been our guiding principles for characterizing the *ACT2:COR* transformants by prioritizing trait assessments over expression analysis. For these reasons, we decided to drop the use of "overexpression" from the current manuscript and opted for "constitutive" or "ectopic" *COR* expression instead.

Reviewer #1

This study by Ortega et al. utilized a bacterial SA synthase gene IRP9 to drive high level of SA in Arabidopsis to circumvent complex nature of defense response induction seen in autoimmune mutants. The resulting hiSA lines exhibited growth defects in a SA dosage dependent manner. The authors also identified a group of COR genes whose expression are inhibited by SA. Most strikingly, overexpressing three COR genes reversed the growth defects of the hi-SA plants.

It is well known that the plant defense hormone salicylic acid (SA) inhibits plant growth, but its underlying molecular mechanism is not fully understood. This study provides a possible link, that is COR gene expression, between SA and growth inhibition. It suggests that defense response and growth inhibition can be potentially uncoupled, which could lead to finer manipulation of defense responses without adversely affecting plant growth. This finding, when further substantiated, would be significant to the field of plant-growth interaction.

The paper is well written. Experiments are well designed in general. Methods are explained in great detail. For instance, co-suppression was described although these lines were not reported. Data is in general of good quality.

We appreciate the reviewer's encouraging remarks. We have now included data from the cosuppression experiments, since they provide relevant information and serve as additional replications for the observed COR phenotypes.

Below are a few main concerns when addressed would substantiate the conclusion greatly.

1. Characterization of the hiSA COR-OE lines.

1) More lines need to be characterized. Now only two lines are shown although it is said that they were randomly selected. Additional lines could be looked at, but they do not need to be as thoroughly analyzed as the 2 existing lines.

Images for additional *ACT2:COR* events have been included in the new Fig. S5, showing improved growth during tissue culture T_1 selection (one per background for *COR15a*) and in soil (one per construct for WT, F24 and *siz1-2* background). For in-depth characterization, we presented data for 3 ACT2:COR constructs (COR15a, COR15b, COR6.6) in 5 genetic backgrounds (WT, F24, F31, F36, and *siz1-2*) for a total of 15 independent transformation trials and 30 independent lines. This represents a high degree of reproducibility for the observed COR rescue of hiSA plant growth. As noted above, we also included data from the *35S:COR* transformants to further support the reproducibility of COR phenotypes across multiple generations regardless of *Fd-Irp9* silencing.

2) Expression of IRP9 and COR genes should be analyzed in hiSA COR-OE lines and compared to hiSA lines and COR-OE lines. This is because hiSA COR-OE were not created by crossing of a hiSA line with a COR-OE line and therefore the expression may not be comparable.

As explained on p. 1-2 above, we prioritized SA measurements and Pst DC3000 disease resistance over gene expression analysis, in part because of the visually distinct COR phenotypes (leaf expansion and senescence), and because of prior experience with the "cosuppression" group (new Fig. S4 and Fig. 5). We detected similar *COR15* transcripts levels in the T_4 *35S:COR* transgenics of both F24 (which exhibited *Irp9* silencing) and WT backgrounds, but none exceeded those of NahG plants to qualify as "overexpression". Regardless, all *35S:COR* transgenics (3 constructs, 2 backgrounds, 2 lines = 12 total) exhibited prolonged leaf expansion and delayed senescence. These phenotypes were also observed in *ACT:COR* transgenics, altogether 44 independent events (3 constructs, 5 backgrounds, 2 lines = 30 total shown in Fig. 6 and Fig. S6, plus 14 additional events shown in Fig S5). We reasoned that the COR phenotypes were driven by ectopic/constitutive *COR* expression rather than "overexpression" *per se*. We have changed the manuscript text to describe *ACT:COR* transgenics as ectopic expression (*eCORs*) and dropped the use of OE/overexpression. We presented LC-MS data to show no apparent loss of SA accrual in the T_2 *eCOR* lines and performed Pst Dc3000 assays in homozygous lines to demonstrate no loss of disease resistance in the F24 and F31 background. Unfortunately, we are not in position to repeat the plant growth experiments to collect gene expression data due to funding and project personnel moving on. However, the high number of transgenic lines from multiple independent transformations demonstrates highly reproducible COR phenotypes similar to previously reported (see below).

2. Growth promoting phenotype of COR overexpression. Previous study did not find a growth promoting activity of COR genes. In fact, COR15 overexpression was reported to have slight growth inhibition (Thalhammer and Hincha, 2014). This discrepancy can be addressed. In particular, is this a 16C specific effect at least in wild type, as indicated by sFig 3b? COR genes are induced by a low temperature. Why would an overexpression of COR increase growth at 16C? Expression of COR genes can be analyzed in different COR-OE lines at both temperatures.

The *COR15a* and *COR15b* overexpressors described in Thalhammer et al. (2014) were generated and first reported by Yang et al. (2011), which has been a key reference in our work. No growth penalty of

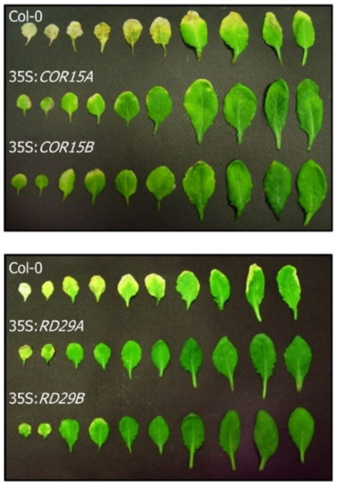

Fig. 7C-D from Yang et al. (2011) showing delayed senescence and prolonged leaf expansion of 35S:COR transgenics.

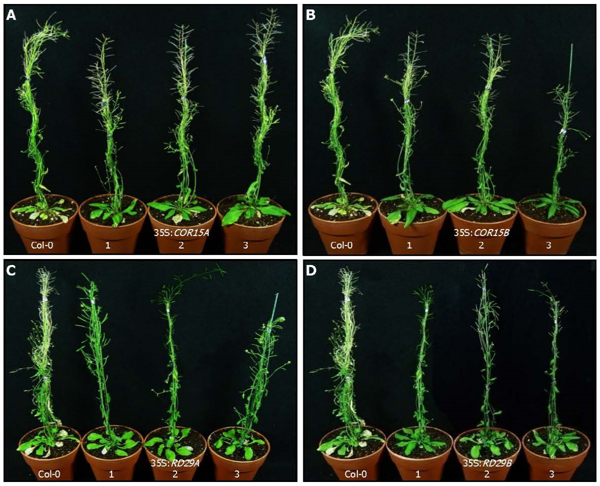

Fig. S16 from Yang et al. (2011) showing whole-pla[illegible] delayed senescence and prolonged leaf expansion [illegible] transgenics.

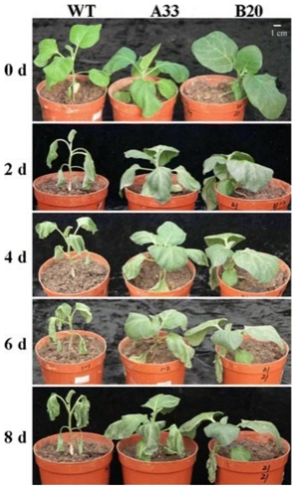

Fig. 1 of Wan et al. (2014) showing increased expansion of eggplant leaves expressing *AtCOR15a* driven by *AtRD29a* (*COR78a*) promoter (line B20 at 0 d). The unrelated A33 was from *AtRD29a:AtCBF3*.

COR15-OE (and COR78/RD29-OE) plants was mentioned in the original paper. In fact, they highlighted prolonged leaf expansion and delayed senescence similar to what we observed in our *35S:COR* and *ACT2:COR* transgenics (key figures from Yang et al. pasted below for your information). A similar phenotype (larger leaves) was also seen in transgenic eggplants overexpressing *COR15A* (Wan et al., 2014), also shown below. In these two papers, the phenotypes were observed at ambient temperatures.

Thalhammer et al. (2014) obtained seeds of *COR15*-OE lines from Yang et al. (2011) for their work. We could only speculate that the slight growth inhibition in their paper was due to variation between lab-adapted Col-0 WT. We believe data from the original paper described above were more appropriate reference for our work. We did reference multiple papers from Thalhammer, Hincha and colleagues on their biophysical and molecular dynamics modeling studies on COR and LEA proteins.

In our study, the COR effects are observed at both ambient and subambient temperatures in *35S-COR* (Fig. 5, Fig. S4) and *ACT2:COR* (Fig. 6, Figs. S5-S6) transformants. We are reluctant to describe the COR phenotypes we observed (delayed senescence and prolonged leaf expansion) as "growth-promoting" *per se*, in part because the effect on biomass varies depending on genotype, growth condition, and plant age. The growth improvement of *COR*-transformants is much more striking under growth-limiting scenarios, such as in hiSA or *siz1-2* backgrounds vs. WT (at both 22°C and 16°C) and at 16°C vs. 22°C for all backgrounds when growth is slowed by lower temperatures. The latter undoubtedly reflects the multiple functions of CORs, including their originally reported role in cold tolerance (Thomashow, 1999) as well as their involvement in leaf senescence and expansion (Yang et al., 2011). We have significantly revised the discussion to address these points. The involvement of transcriptional circuits in the SA inhibition of *CORs* requires further research and this is also covered in the revised discussion.

3. Quantification of growth defect.
The rosette size is not so different between hiSA lines (such as F24 and F31) and wt in picture (Fig. 4a), but the dry weight was 2-fold difference (Fig. 4b). Does that mean that fresh weight is not as different between the wt and the transgenic plants?
It would be good to quantify the fresh weights (which is the more commonly used in other growth-defense studies) so that the study can be more comparable to previous studies.
In addition, if hiSA COR-OE lines and the COR-OE lines differ in fresh weight (dry weight appeared to be same), it would be good to determine the interaction of effects from hiSA and from COR-OE to support the conclusion that COR-OE suppresses growth defect of hiSA rather than acting in an independent pathway of growth promotion.
To clarify, the hiSA plants have significantly lower rosette biomass than the WT, more strikingly so at 16°C than 22°C (now Fig. 6A-D). The results were reproducible as shown also in Fig. 1D (22°C), Fig. 4C and replicate data in Fig. S3B (22°C and 16°C), and Fig. S4E-G (22°C and 16°C). We have now included rosette canopy diameter and rosette thickness in Fig. 1D and Fig. S4E to show that hiSA plants have reduced rosette area and thickness, culminating in reduced biomass.

We collected fresh weight and dry weight data for some experiments, including for hiSA lines (now in Fig. S3) and the *35S:COR* (co-suppression) group which also included hiSA lines (now in Fig. S4). In both cases, the moisture content did not differ between genotypes at either temperature. For this reason, we only collected dry weight data for the *ACT2:COR* plants.

4. SA-dependent growth defect as in L204. The exacerbated growth defect at 16C compared to 22C is stated as a SA dosage dependent. Although this is true among different lines at one temperature, SA accumulation is lower in 16C compared to in 22C while growth defect is more severe (Fig. S2). The

exacerbated growth defect is therefore not from an increase of SA accumulation at 16C. This needs to be discussed. Is this more defense amplification or more inhibition of COR expression at low temperatures?
We agree and it's the latter (more *COR* inhibition at low temp). We have significantly revised the discussion to clarify this point. We ascribed the exacerbated growth defect of hiSA lines at 16C to compromised COR protection due to SA suppression of *COR* induction. Under non-stress conditions, *COR* expression is much lower and not as sensitive to elevated SA compared to the *COR* levels detected at cool temperatures (much more abundant and stronger SA suppression, as shown in Fig. 4F). We have revised the model (now Fig. 7) to depict the dynamic relationship between SA and COR in response to biotic and abiotic cues.

Additional points to consider:
1. Lines 63-74. Autoimmune mutants have various origins and there are many of them. It is good to cite a review or two to introduce autoimmune mutant comprehensively. SA accumulation is a usual feature, but activation of NLR genes is also common (Freh M, et al, 2022 Plant Physiol 188:1419). hiSA lines are not equivalent to autoimmune mutants where additional aspects of defense responses might be activated.
We have included new references on autoimmune mutants. We agree that hiSA lines are not equivalent to autoimmune mutants as supported by our data.

2. L188-192. Th genes listed, CPR5, DND1, SIZ1 and SRFR1, while their mutations lead to autoimmune, were not reported (or not relevant) to have their RNA expression changed upon SA induction in autoimmune mutants.
Agreed. We deleted this sentence.

Reviewer #2
Ortega and colleagues have reported an exciting discovery of the molecular basis of Arabidopsis plant growth-defense trade-off mediated by the hormone salicylic acid. This is an important manuscript with implications in plant hormone signaling, abiotic stress tolerance, plant immunity and gene regulation. A major strength of this study is the successful implementation of the bacterial enzyme-based orthogonal approach (originally developed in poplar - Xue et al. 2013 Plant Cell; doi.org/10.1105/tpc.113.112839) in overproducing SA in Arabidopsis. This bypassed the pleiotropic effects of previously reported SA-overaccumulating mutants. In detail, the authors report that SA overaccumulation expectedly leads to growth defects in Arabidopsis, which is associated with upregulated in SA/SAR-related genes (as expected). Remarkably, they find that SA overaccumulation leads to downregulated expression of a suite of COR genes. The authors then "rescue" COR transcript levels by transgenic overexpression in their high SA mutants, which then boosts plant growth without sacrificing immunity to the hemibiotrophic pathogen P. syringae DC3000. In addition to this major conceptual advance, this study provides numerous plant materials (transgenic lines) and global transcriptome datasets to the research community. Finally, this exciting discovery could pave the way for potential applications in bypassing growth-defense trade-offs in crop plants - a holy grail in the plant sciences.

Because of the broad implications of this study, the paper needs to properly show strong evidence to support the stated conclusions. It is also important to exercise caution that certain aspects of plant defences and growth still need to be investigated in future studies. Specifically:
We appreciate the reviewer’s encouraging remarks and have reworked the discussion, including the need for further research into the SA-COR regulation.

1) The major thesis of this paper is that COR overexpression does not interfere with SA production and disease resistance in the high SA lines (F24 and F31) under different temperatures. However, there is no Pst DC3000 data in Figure 4 showing that disease resistance was maintained with COR overexpression at 16°C. In parallel, there is no SA data in Figure S3 showing that hormone levels are unchanged after COR overexpression at 22°C.
We have now added representative data as requested. We measured SA levels in randomly selected *ACT2:COR* T_2 plants (one plant per transgenic line) at 22°C to confirm no apparent loss of SA accrual (this data is now included in Fig. S6A). We prioritized replicated sampling from the T_3 population for *Pst* DC3000 disease assay (Fig. 6F). We hope the reviewer can appreciate the scope of the experiment, with numerous genotypes (5 backgrounds, 3 transgenes, 2 events each, 2 temperatures) and phenotypes to measure in limited growth chamber space.

For 16°C experiments, we have already performed multiple trials with hiSA lines (plus the *35S:COR* transformants) to show no major changes in SA accrual between 22°C and 16°C (this was noted by reviewer 1's comment 4 above). Thus, we have no reason to expect changes in disease resistance. In practice, it is extremely difficult to perform disease assays by syringe infiltration using 16°C plants because their leaves are much thicker and frequently curled (see Fig. S4E and Fig. 6B). While *in vitro* seedling flood inoculation works well in our hands under normal tissue culture conditions, plant growth at 16°C was much more variable among biological replicates due to frequent occurrence of condensation inside the petri dishes. Nevertheless, we performed the disease assays by seedling flood-inoculation at 16°C and observed more robust growth of F24 and its *ACT2:COR* derivatives than the WT. We have included representative images from replicate petri dishes (Fig. 6G and Fig S6C). Unfortunately, our attempt to quantify bacterial growth from that experiment was not successful due to personnel turnover and miscommunication.

Two other studies also reported limited changes of SA levels between 22°C and 16°C (Li et al., 2020; Bruessow et al., 2021). They performed disease assays across a number of ecotypes and mutants by dipping and showed similar *Pst* DC3000 growth response trends at 22°C and 16°C (Li et al., 2020; Bruessow et al., 2021).

2) As a follow-up to #1, the authors overexpressed three different COR genes in their high SA mutant lines (as shown in Figure 4). Can the authors show confirmation of COR overexpression by providing COR gene expression data in their lines?
As explained on page 1 and in our response to reviewer 1, #2, we prioritized our efforts toward trait characterization rather than expression measurements. This was due to prior experience with *35S:COR* plants which exhibited prolonged leaf expansion and delayed senescence (COR phenotypes) even though *COR* expression was below that detected in NahG plants (Fig. S4J). We have replaced "overexpression" with "ectopic expression" in the manuscript. Our data show reproducible *COR* phenotypes similar to those originally reported by Yang et al. (2011) in 12 different *35S:COR* transgenic lines (plus various F1 and F2 crosses) and 30 *ACT2:COR* transgenic lines (plus 12 additional lines) across multiple genetic backgrounds and at two different growth temperatures.

3) The paper shows that any of the COR genes tested could rescue the growth of high SA mutants without significant impacts on disease resistance. This is an intriguing finding that brings forth new questions. Why is it that a single COR gene is remarkably sufficient to uncouple this longstanding trade-off? The authors reason that their "results align with the notion that cellular robustness encompasses

both functional redundancy and specificity" (Lines 265-266). Perhaps this can be more fleshed out in the Discussion section. Even speculation on potential mechanisms based on crosstalk between SA and growth hormones (BR, IAA, etc.) would be insightful to readers.
We expanded on that point with significant revision to explain that functional redundancy can be conferred by duplicated genes (e.g., *COR15a* and *COR15b* tandem duplicate) or unrelated genes involved in alternative pathways (e.g., different *CORs* or *LTIs*). A defining feature of redundant genes is their ability to compensate for each other's loss. Based on the overlapping functions of *COR* genes (shared inducibility by multiple dehydration stresses and inhibition by SA), it is not unexpected that any one of the *COR* genes could compensate for losses of multiple *CORs* caused by SA suppression to restore hiSA plant growth.

As part of the discussion overhaul, we also cited the study of Campos et al. (2016) as another successful example of decoupling the growth-defense tradeoff, in this case, involving JA. The two examples share a striking analogy as both dealt with negative interactions among intersecting signal transduction pathways (JA signaling and shade avoidance in Campos et al., and SA signaling and COR response in our study). We offered some speculation and pointed out the need for further research to investigate other hormonal pathway crosstalk.

4) This is more philosophical but it would be great for the authors to highlight in the Discussion section why plants even evolved growth-defense trade-offs if simple COR overexpression is sufficient to uncouple this trade-off. Are there any hidden trade-offs of COR overexpression in plants? Are there natural instances (in certain Arabidopsis accessions) where the SA-growth trade-off is not apparent?
We view the various tradeoffs as consequences of constantly evolving defense strategies under varying conditions. We touched upon some of these points in the revised discussion to address this as well as comments #3 above and #5 below. We speculated on the most obvious tradeoff between SA inhibition of CORs as to restrict leaf longevity (i.e., premature senescence) upon activation of SA defense to optimize reproductive success.

Motivated by the reviewer's question about the tradeoff in natural accessions, we mined published data to further explore the SA-COR relationship. The majority of the 100 genes negatively regulated by SA identified from Fig. 3A was also classified as "SA-negative" in a meta analysis (Zhang et al., 2020). Analysis of Col-0 and C24 by Miller et al. (2015) also supports a negative relationship between SA and *COR* expression, as *COR* transcript levels were one to two orders of magnitude higher in Col-0 than in C24, with the latter known to accumulate higher levels of SA. These points have been added to the discussion. We also mentioned the need for further research into the SA-COR regulation and other hormonal crosstalk.

5) As a follow-up to #4, cold temperatures are known to induce both SA production and COR gene expression. So, why is it that growth suppression at this temperature is still observed? It may be good to discuss cold temperature regulation of plant growth that is SA-independent.
In response to this comment, we have revised the model in Figure 7 and Discussion. In short, the growth behavior at different temperatures reflects the net outcome of the dynamic SA and COR responses to external cues. Under our experimental conditions, cool temperatures significantly induced *COR* expression by multi folds, but SA levels were largely unchanged as mentioned above. The low temperature-induced COR protection was canceled out by SA hyperaccumulation in hiSA lines, resulting in exacerbated growth penalty. We added two paragraphs to discuss the SA-COR responses to cold/cool temperatures and their opposing effects on leaf senescence and longevity. We also included published data in support of the negative regulation of *CORs* by SA as mentioned above.

Minor comments:
1) Lines 60-61: It would be great to cite the seminal papers that postulated the growth-defense trade-off principle.
done

2) Lines 68-70: Apart from being an RNA-binding protein, CPR5 is also a component of the NPC (Gu et al., Cell; https://doi.org/10.1016/j.cell.2016.07.042). It would be great to at least acknowledge this.
both roles are now explained.

3) Lines 63-64: Because the authors mention the dnd1 and srfr1 mutants in the first paragraph, it would be great to briefly provide their biological importance in this paragraph.
done

4) Lines 106-108: It is stated that Line F24 "had SA derivative levels that approached or surpassed those detected in the autoimmune mutants siz1-2 and dnd1." However, Figure 1b shows barely noticeable SAG/DHBX and SGE increases in F24. Based on this data, F24 resembles F19. I may have missed something, but can the authors please clarify this?
The statement was based on our overall assessment of the hiSA lines over multiple generations and many replicate experiments of homozygous lines. We replaced the Fig. 1 SA metabolite panels with data from T_4 plants that are more representative of the overall trends, and added a new Fig. S1 for SA data from T_1 through T_3 (which was previously used for Fig. 1). Additional data from autoimmune mutants were included when available (*siz1-2* and *cpr5-2* in Fig 1, *siz1-2*, *dnd1*, *cpr5-2* in Fig. S1). Repeated SA measurements (of hiSA lines) were shown in Fig. 5B,E (22°C and 16°C), Fig. S3 (22°C and 16°C, with *srfr1-4*), Fig. S4H (22°C and 16°C), and Fig. S6A (22°C, with *siz1-2*). Despite occasional variation, the statement cited above still holds.

5) Lines 168-171: Apart from the NPR receptors and transcriptional coregulators, did the transcriptome also show upregulated expression of major SA-associated transcription factors, e.g. CAMTA1/2/3, SARD1 and CBP60g? How about PTI and ETI-related genes? Showing this data in Figure 2a may help broaden the paper's findings.
The upstream TFs were not significantly changed in hiSA lines, with the exception of SARD1 which was positively regulated by SA. SARD1 has been added to the zoom-in group of Fig 2A. None of the PTI (BIK1, BAK1, EFR, FRK1, MPK3, MPK6, MIN7) or ETI (NDR1, RAR1, RIN4, RPM1, RPS2) markers were significantly changed in hiSA lines, except FLS2 and CERK1 that were altered only in the two extreme genotypes NahG (reduced) or F51 (induced). These are now added to the text and Fig 2A.

6) Lines 243-244: "Constitutive COR gene expression also rescued the growth defect of siz1-2 at both temperatures." - Based on the data in Figure 4b and Figure S3, I think this may be a partial rescue since the rosette dry weight does not approach that of the wild-type plants.
We agreed and have changed the wording. We noted slight differences between the three COR groups (especially COR6.6 at 22°C), presumably reflecting their overlapping-yet-distinct functions as mentioned in the manuscript.

7) Line 250: I feel like this paragraph ends abruptly and could be missing a concluding sentence.
We added a wrap up sentence at the end.

8) Lines 268-269: The statement on COR gene phylogeny needs a citation.

We have revised the discussion and simplified the description of the COR family to replace the original sentence.

9) Line 323: Clarity the primers used to confirm the transgenics.
done (*Irp9* and *HPT*). Similar info was added to other transgenics.

10) Line 355: Could the authors state the approximate number of times that the experiment was repeated with reproducible results?
We added a new section on growth monitoring and biomass analysis with more details since growth response is a major component of the work. The hiSA lines have been examined at least 10 times over multiple years with reproducible patterns, including as the reference group for *35S:COR* or *ACT2:COR* transgenics. We also included repeat info for other experiments throughout the Methods.

11) Line 358, Lines 379-380 and Line 418: How old were the plants? Please clarify.
We added the info to the Methods. Metabolite and gene expression analyses of soil-grown plants were done at bolting, or stage 5.1 based on Boyes et al. (2001). We pooled 3 mature leaves per plant during sampling, which was sufficient for both RNA extraction and metabolite analyses in some experiments.

Disease assays were also performed at bolting (small experiments) or 1-2 days before/after large-scale sampling for logistics reason.

12) Lines 384-385: Indicate the supplies for the HPLC-MS standards used.
We revised the text to add supplier information for commercially available standards and citation for two previously confirmed compounds. We also included our strategy for confirmation of additional conjugates published previously by others and added a new supplemental figure (Fig. S2) with supporting LC-MS data.

13) For Figures 1,3, 4 and S1 to S3: please clearly define n (Are they individual plants? Single pools of several plants? Individual leaves of the same plant?, etc.). In addition, please indicate the number of reproducible independent experimental repeats performed.
We stated the sampling process in the Methods. All analyses were done using individual plants as biological replicates, except for *in vitro* assays when plants/seeds were pooled as one biological replicate. We have now added the info to all figure legends as well. Information on the experimental repeat numbers was provided in the Methods, and now included in the legends.

14) This may be a minor issue but it seems unconventional for the panel letters to not be chronologically arranged in Figures 1 and 3.
We have split the old Figure 1 into two (new Figs 1 and 2) and rearranged panels in old Figure 3 (now Fig. 4) to avoid this issue.

Boyes, D.C., Zayed, A.M., Ascenzi, R., McCaskill, A.J., Hoffman, N.E., Davis, K.R., and Görlach, J. (2001). Growth stage-based phenotypic analysis of Arabidopsis: A model for high throughput functional genomics in plants. Plant Cell **13,** 1499-1510.

Bruessow, F., Bautor, J., Hoffmann, G., Yildiz, I., Zeier, J., and Parker, J.E. (2021). Natural variation in temperature-modulated immunity uncovers transcription factor bHLH059 as a thermoresponsive regulator in Arabidopsis thaliana. PLOS Genetics **17,** e1009290.

Campos, M.L., Yoshida, Y., Major, I.T., de Oliveira Ferreira, D., Weraduwage, S.M., Froehlich, J.E., Johnson, B.F., Kramer, D.M., Jander, G., Sharkey, T.D., and Howe, G.A. (2016). Rewiring of

jasmonate and phytochrome B signalling uncouples plant growth-defense tradeoffs. Nat Commun **7,** 12570.

Li, Z., Liu, H., Ding, Z., Yan, J., Yu, H., Pan, R., Hu, J., Guan, Y., and Hua, J. (2020). Low temperature enhances plant immunity via salicylic acid pathway genes that are repressed by ethylene1. Plant Physiology **182,** 626-639.

Miller, M., Song, Q., Shi, X., Juenger, T.E., and Chen, Z.J. (2015). Natural variation in timing of stress-responsive gene expression predicts heterosis in intraspecific hybrids of Arabidopsis. Nature Communications **6,** 7453.

Thalhammer, A., Bryant, G., Sulpice, R., and Hincha, D.K. (2014). Disordered Cold Regulated15 proteins protect chloroplast membranes during freezing through binding and folding, but do not stabilize chloroplast enzymes in vivo. Plant Physiology **166,** 190-201.

Thomashow, M.F. (1999). Plant cold acclimation: Freezing tolerance genes and regulatory mechanisms. Annual Review of Plant Physiology and Plant Molecular Biology **50,** 571-599.

Wan, F., Pan, Y., Li, J., Chen, X., Pan, Y., Wang, Y., Tian, S., and Zhang, X. (2014). Heterologous expression of Arabidopsis C-repeat binding factor 3 (AtCBF3) and cold-regulated 15A (AtCOR15A) enhanced chilling tolerance in transgenic eggplant (Solanum melongena L.). Plant Cell Reports **33,** 1951-1961.

Yang, S.-D., Seo, P.J., Yoon, H.-K., and Park, C.-M. (2011). The Arabidopsis NAC transcription factor VNI2 integrates abscisic acid signals into leaf senescence via the COR/RD genes. Plant Cell **23,** 2155-2168.

Zhang, N., Zhou, S., Yang, D., and Fan, Z. (2020). Revealing shared and distinct genes responding to JA and SA signaling in Arabidopsis by meta-analysis. Frontiers in Plant Science **11**.

Dear Dr. Chung-Jui Tsai:

We have received reviews of your manuscript entitled "Decoupling the salicylic acid-growth tradeoff by altering cold-regulated gene expression in Arabidopsis." On the basis of the advice received, the board of reviewing editors would like to accept your manuscript for publication in The Plant Cell.

This acceptance is contingent on revision based on the comments of our reviewers. In particular, please consider the comments by Reviewer #2, and add to the discussion the points they have raised on including new literature on ICE1, and discuss the the expression levels of COR genes in the sid2 background, in the light of a previously published study.

Please highlight all changes and include a detailed annotation of changes of the text, with line numbers, and noting your responses to the comments.

To submit your revised manuscript, click:

Link Not Available

If you have any questions about the revision submission procedures, please contact the Editorial Office Staff (tpc-submissions@aspb.org). If you cannot return the revised manuscript within 30 days, please let us know. Otherwise, we will assume that you have elected not to revise the manuscript and withdraw it.

Thank you very much for the privilege of reviewing this work. I look forward to receiving the next version.

On behalf of the editorial board,

Cris Argueso, Senior Editor
Blake Meyers, Editor-in-Chief

The Plant Cell

Reviewer #1 (Comments for the Author):

The manuscript is greatly improved after revision. New data are provided to substantiate the conclusions. Co-suppression is resolved with a new set of transgenic plants. In addition, the discussion is re-written to synthesize results from this study and knowledge in literature, giving some new insights on the function of SA and COR genes in plant growth and senescence. The story has now more clarity conceptually. A minor suggestion is on the model. Figure 7 is meant to depict three scenarios, but the way A, B, C are used makes them look like three independent panels.

Reviewer #2 (Comments for the Author):

Dear Authors/Editor,

Ortega and colleagues have conducted a comprehensive revision of their manuscript, further strengthening the experimental evidence to support the authors' conclusions. The amount of work in performing additional experiments to address the reviewer's suggestions and concerns should be highly commended!

Clarifications/comments:

Lines 275-276: I suggest being cautious in the wording that some of the rescued phenotypes are partial. That is, certain COR-expressing Hi-SA plants do not reach the growth phenotypes of Col-0 plants similarly expressing the COR genes. I would suggest adding this context within this paragraph.

Lines 294-295: Similar to my comment above, I suggest toning down "...exhibited essentially no growth penalty..." to "...exhibited reduced growth penalty..." to take into consideration the partial phenotype of the COR6.6-expressing Hi-SA lines.

Lines 330-331: I suggest stating that this is a partial (and not full) rescue of growth inhibition by COR transcriptional rewiring.

Discussion Point #1: I would suggest incorporating the following studies since the ICE1 transcription factor (which directly regulates COR genes) has recently been implicated in SA signaling at low temperatures: Li et al., 2024 Plant Cell

(https://doi.org/10.1093/plcell/koae096); Tang et al., 2020 JIPB (https://doi.org/10.1111/jipb.12918).

Discussion Point #2: Can the authors please reconcile their current conclusions with the previous findings in the Kim and Thomashow et al. 2013 Plant Journal paper (https://doi.org/10.1111/tpj.12205)? In that study, Kim and colleagues showed using transcriptome sequencing that COR genes (e.g. COR15a, COR15b, COR6.6) are not differentially regulated between wild-type Col-0 plants (which induces SA at low temperature) and SA-deficient sid2-1 mutant plants (which cannot induce SA at low temperatures). If SA always suppresses COR gene expression, shouldn't one expect that SA-deficient sid2-1 plants would have increased COR transcript levels? Although new experimentation is not needed, a textual clarification by the authors in the Discussion section would be recommended.

Please also note the following:

-The Plant Cell now requires authors to complete and submit an author revisions checklist upon submission of a revised manuscript. The aim of the checklist is to aid authors in preparing a high-quality manuscript, facilitate the review and assessment of revised manuscripts, and help to ensure that journal standards are maintained across the board. If your manuscript is accepted, the completed checklist will be published as supplemental material attached to the article online. Please download a copy of the checklist (pdf fillable form) at this link, for submission with your revised manuscript: https://tpc.msubmit.net/html/Author_Revisions_Checklist.pdf.

-Supplemental materials should be restricted to large datasets and tables, presentation of replicates, and validation of reagents, methods, or genotypes. Any data that are used to support the major claims must be in the main manuscript. Supplemental figure legends must indicate what figure in the main manuscript is supported by the supplemental data presented. Please justify how each of the supplemental figures meet the criteria.

-Sampling methods and the nature of "biological replicates" should be described precisely (i.e. different plants, parts of plants, pooled tissue, independent pools of tissue, sampled at different times, etc.), along with a clear description of and rationale for any statistical analyses conducted. The reader should know exactly what was sampled; what forms the basis of the calculation of any means and statistical parameters reported. This is also necessary to ensure that proper statistical analysis was conducted.

Reviewer #1

The manuscript is greatly improved after revision. New data are provided to substantiate the conclusions. Co-suppression is resolved with a new set of transgenic plants. In addition, the discussion is re-written to synthesize results from this study and knowledge in literature, giving some new insights on the function of SA and COR genes in plant growth and senescence. The story has now more clarity conceptually. A minor suggestion is on the model. Figure 7 is meant to depict three scenarios, but the way A, B, C are used makes them look like three independent panels.

As suggested, we removed the labeling for the three scenarios, now all in panel A.

Reviewer #2

Ortega and colleagues have conducted a comprehensive revision of their manuscript, further strengthening the experimental evidence to support the authors' conclusions. The amount of work in performing additional experiments to address the reviewer's suggestions and concerns should be highly commended!

Clarifications/comments:

Lines 275-276: I suggest being cautious in the wording that some of the rescued phenotypes are partial. That is, certain COR-expressing Hi-SA plants do not reach the growth phenotypes of Col-0 plants similarly expressing the COR genes. I would suggest adding this context within this paragraph.

We feel that the tone in this paragraph was soft, describing the potential to “alleviate” growth inhibition by severing SA suppression of COR genes. We made the suggested changes below.

Lines 294-295: Similar to my comment above, I suggest toning down "...exhibited essentially no growth penalty..." to "...exhibited reduced growth penalty..." to take into consideration the partial phenotype of the COR6.6-expressing Hi-SA lines.

Done

Lines 330-331: I suggest stating that this is a partial (and not full) rescue of growth inhibition by COR transcriptional rewiring.

We changed this to “fully or partially rescued” to more accurately reflect our findings.

Discussion Point #1: I would suggest incorporating the following studies since the ICE1 transcription factor (which directly regulates COR genes) has recently been implicated in SA signaling at low temperatures: Li et al., 2024 Plant Cell (https://doi.org/10.1093/plcell/koae096); Tang et al., 2020 JIPB (https://doi.org/10.1111/jipb.12918).

We included ICEs in the results of the first RNA-Seq data when we first introduced CBFs. However, expression of ICEs was unaffected in hiSA lines or by cooling in the present study. This has now been added to the discussion. In brief, neither ICE1 nor its interacting partners NPR1 and TGA3 were transcriptionally affected by SA or cooling.

Discussion Point #2: Can the authors please reconcile their current conclusions with the previous findings in the Kim and Thomashow et al. 2013 Plant Journal paper (https://doi.org/10.1111/tpj.12205)? In that study, Kim and colleagues showed using transcriptome sequencing that COR genes (e.g. COR15a, COR15b, COR6.6) are not differentially regulated between wild-type Col-0 plants (which induces SA at low temperature) and SA-deficient sid2-1 mutant plants (which cannot induce SA at low temperatures). If SA always suppresses COR gene expression, shouldn't one expect that SA-deficient sid2-1 plants would have increased COR transcript levels? Although new experimentation is not needed, a textual clarification by the authors in the Discussion section would be recommended.

This point has already been addressed in the results (Fig. 4F) and discussion (3rd paragraph, 3rd sentence):

Results (p. 9): “Indeed, transcript levels of all leaf-expressed COR genes were significantly higher at 18°C than at 22°C in all three genotypes (Fig. 4F). However, the magnitude of COR gene induction was attenuated by SA, being lowest in F24 and highest in NahG (Fig. 4F).”

Discussion (p. 12): “Cooling stimulated PR gene expression in an SA-dependent manner, but SA is not required for cooling-induced *COR* expression (Wang and Hua, 2009; Kim et al. 2013) (Fig. 4F). Instead, we show that SA had a detrimental effect on the induction of COR and other low-temperature sensitive genes (Fig. 4F).”

We added Kim et al. 2013 reference to the discussion. We note that Kim et al. 2013 used microarray which might explain its lack of sensitivity in picking COR induction differences between WT and sid2-1 as we have shown for WT and NahG using RNA-Seq. It is also possible that the residual SA levels in sid2-1 via ICS2 or PALs (Wu et al., 2019) could have attenuated the difference between WT and sid2-1 in Kim et al. 2013. Because our data directly support the Reviewer’s expectation (greater COR induction in SA-deficient NahG plants), we do not feel it is necessary to expand the discussion to explain another study that used a different mutant (sid2-1, which is not truly SA-deficient), different stress treatments (cold), and an older and less sensitive gene profiling method.

Wu, Z., Han, S., Zhou, H., Tuang, Z.K., Wang, Y., Jin, Y., Shi, H., and Yang, W. (2019). Cold stress activates disease resistance in Arabidopsis thaliana through a salicylic acid dependent pathway. Plant, Cell & Environment **42,** 2645-2663.

13-Jun-2024

Dear Dr. Chung-Jui Tsai:

We are pleased to inform you that your paper entitled "Decoupling the salicylic acid-growth tradeoff by altering cold-regulated gene expression in Arabidopsis" has been accepted for publication in The Plant Cell, pending a final editorial review by a science editor. At this stage, your manuscript will be evaluated by a science editor with respect to its presentation of scientific content, compliance with journal policies, and presentation for a broad readership. The Plant Cell has contracted with Plant Editors (planteditors.com) to provide this service to our authors, and you will soon receive additional information on this process.

Please note that each author needs to link their ORCID identifier to their account before your manuscript can be published. If any authors do not have an ORCID linked to their account, they will receive a message with a link to complete this task. Please follow up with your coauthors and ensure that they have completed this task as soon as possible.

The Plant Cell and The Arabidopsis Information Resource (TAIR) are collaborating to collect functional annotation data about Arabidopsis genes from authors. This includes information about the gene's molecular function (e.g., kinase activity, ATP synthetase activity), the biological process/es it is involved in (e.g., endosperm development, threonine biosynthesis), its subcellular location (e.g., nucleus, ER), anatomical or developmental expression pattern (e.g., leaf, ovule, flower stage 10, seedling stage), or its partner in a protein-protein interaction (e.g., AT1G01010 interacts with AT1G01020).

If your paper contains results falling into one or more of these categories for Arabidopsis genes, we request that you now submit these data for inclusion in TAIR by filling in the form provided at the following URL: https://www.arabidopsis.org/doc/submit/functional_annotation/123. If you need further clarification on what types of data can be submitted please contact curator@arabidopsis.org.

Finally, we encourage your submission of artwork for the journal cover. Monthly issues will have an online cover image and selected covers will be used for posters, other promotional items, and "wallpaper" for mobile devices. For more information, refer to Cover Submission in the Instructions for Authors https://tpc.msubmit.net/cgi-bin/main.plex?form_type=display_auth_instructions.

We look forward to seeing your paper published.

On behalf of the editorial board,

Cris Argueso, Senior Editor
Blake Meyers, Editor-in-Chief

The Plant Cell

----FOR ASPB OFFICE USE ONLY (DO NOT EDIT)----
MSID: 39439
Corresponding Author: Prof. Tsai
